# Bacterial abundance and co-acclimation in mangrove rhizosphere and non-rhizosphere soils under pyrene stress

**DOI:** 10.3389/fmicb.2025.1661315

**Published:** 2026-02-06

**Authors:** Han Wang, Ali Mohamed Elyamine, Mengtong Liu, Hongge Shi, Rong Wang, Hongzhou Zhang, Junfeng Qi, Wansen Li

**Affiliations:** 1 College of Biological and Food Engineering, Huanghuai University, Zhumadian, China; 2 Department of Life Science, Faculty of Science and Technology, University of Comoros, Moroni, Comoros; 3 Zhumadian Academy of Agricultral Sciences, Zhumadian, Henan, China; 4 Zhumadian Tobacco Company of Henan Province, Zhumadian, Henan, China; 5 Flavors and Fragrance Engineering & Technology Research Center of Henan Province, College of Tobacco Science, Henan Agricultural University, Zhengzhou, China; 6 College of Computer and Artificial Intelligence, Huanghuai University, Zhumadian, China

**Keywords:** coastal mangroves, bacterial abundance, nutrients availability, acclimation, rhizosphere

## Abstract

In mangrove ecosystems, research on bacterial abundance in the rhizosphere and the non-rhizosphere soils remains limited. Moreover, the variation in bacterial taxonomy during the acclimation of sediment samples subjected to high-molecular-weight (HMW) organic pollutant stress remains poorly understood. This study was conducted in both rhizosphere and non-rhizosphere soils at depths ranging from 0 to 20 cm in the coastal mangrove of Yunxiao to evaluate the diversity and abundance of the bacterial community and to characterize the profile of its variation arising during acclimation under pyrene stress. Rhizosphere sediments were defined as those directly adhering to the roots of mangrove plants, while non-rhizospheres were those collected 3 m away from the roots. Each sample was divided into two groups: the first group was stored at 4 °C for the determination of the physicochemical characteristics of the sediments, and the second group, used for DNA analysis, was stored at −20 °C. A DNA isolation kit was used to extract total genomic DNA from the samples before and after acclimation. Polymerase Chain Reaction (PCR) amplification of the 16S rRNA genes targeting the V3-V4 region was performed. The results of this study showed that although the physicochemical properties of both rhizosphere and the non-rhizosphere sediments were unevenly distributed, no significant difference in bacterial abundance between the two zones was observed. Moreover, the abundance at 0–10 cm depth was significantly higher in both rhizosphere and non-rhizosphere sediments. The acclimation process revealed that pyrene significantly impacted bacterial community composition and abundance. In total, 23 genera were identified in the first transfer (G1), dominated by *Burkholderia* (23.9% vs. 9.23%), *Rhodobacter* (4% vs. 10.95%), *Bacillus* (11% vs. 10%), *Xanthobacter* (6.82% vs. 7.62%), *Dyella* (4.9% vs. 7%), *Pseudomonas* (6% vs. 7.70%), and *Acinetobacter* (5% vs. 8.63%) in non-rhizosphere vs. rhizosphere samples, respectively. Overall, the findings indicate that bacterial abundance in the rhizosphere and non-rhizosphere of mangrove ecosystems may differ from that in terrestrial plants and that the acclimation of functional bacteria could be an effective means of adapting bacterial communities to environmental disturbances.

## 1 Introduction

Studies on the interaction between rhizospheres and non-rhizospheres of terrestrial plants are much more advanced, as evidenced by several scientific reports (Barea et al., 2013; Jurburg and Salles, 2015; Jitendra et al., 2017). The zone commonly defined as the rhizosphere is a specific area known for its nutrient-rich properties. It is enriched with various nutritive resources by plants through root exudates. On the one hand, resources are directly used by soil microorganisms; on the other hand, they also modify the physicochemical properties of the soil and the substrates for soil microorganisms (Pathan et al., 2015, Luo et al., 2018). The rhizosphere is, therefore, an indisputable ecological niche for the growth and proliferation of microorganisms. It is evident that due to the direct or indirect effects of root exudates, microorganisms are significantly more important in the rhizosphere than in the non-rhizosphere. For example, Picariello et al. (2020) estimated that the microbial population in the rhizosphere of terrestrial plants is approximately 100 times higher than that in the non-rhizosphere (Fan et al., 2017). However, the excretion of root exudates depends not only on plant type, soil properties, pH, air and soil moisture, and nutrient availability but also, above all, on the microbial and plant species present (Zhang et al., 2013; Gao et al., 2019).

Owing to their living conditions and internal physiology, mangrove plant ecosystems are different from terrestrial plants (Wanapaisan et al., 2018). As a result, because the internal physiology of mangroves differs from that of terrestrial plants, the composition of root exudates could also vary and be distinguished from those produced by terrestrial plants. This variation could therefore influence microbial diversity and abundance in both the rhizosphere and the non-rhizosphere of mangrove ecosystems. To date, to the best of our knowledge, few scientific data on variation in microbial populations in the rhizosphere and non-rhizosphere of mangrove plants are available. Luo et al. (2018), studying the influence of mangrove roots on microbial abundance, reported that bacterial abundance was slightly higher in non-rhizosphere sediments, while fungal abundance was markedly higher in rhizosphere sediments (Luo et al., 2018). A metagenomic study comparing the microbial community of *Avicenna marina* mangrove sediments with the bulk soil microbiome found that non-rhizosphere samples had taxonomic and functional profiles similar to those of the rhizosphere (Alzubaidy et al., 2016).

Microorganisms in the rhizosphere, particularly bacteria, can adapt and grow in extreme conditions, including polluted environments (Miura et al., 2019). Bacteria can metabolize and utilize a diverse range of organic pollutants as a source of carbon and energy, including polycyclic aromatic hydrocarbons (PAHs) (Dash et al., 2013). For this reason, PAHs are widely used in both *in situ* and *ex situ* bioremediation techniques for polluted environments. However, research has shown that, in the natural environment, the effectiveness of microbial remediation is greater than that achieved by any single species used individually in *ex situ* conditions, since *in situ*, there is a strong and complex interaction between different microbial communities that live together and promote the process of bioremediation (Passos da Silva et al., 2014). These beneficial interactions in an ecosystem provide a kind of stable balance over a long period of time, which, unfortunately, can be disturbed by the introduction of external stresses and can cause significant changes in the microbial structure (Cho et al., 2012). Scientific reports have shown that the succession of a microbial community in a culture subjected to stress induced by different organic contaminants can be an effective tool for detecting the effect of stress on the microbial community in this medium and for identifying different species, secondary metabolites of the pollutant, and above all, tracing a pathway for the microbial degradation of organic pollutants (Díaz et al., 2013). Although it appears effective, it remains unclear which taxonomic changes may occur upon acclimation to PAH stress. Pyrene is a high-molecular-weight (HMW) PAH, widespread in ecosystems and toxic to humans and the environment. It is one of the most widely used PAHs as a model compound for describing the HMW-PAH degradation pathway (Haihua et al., 2017; Elyamine et al., 2021).

This study was conducted to assess the bacterial community composition in the rhizosphere and non-rhizosphere of mangroves and to evaluate the ability of epiphytic bacteria to tolerate and survive under pyrene stress. As an experimental object, pyrene was used in a culture on mineral salt medium (MSM) as an external stress to assess the ability of microorganisms to survive under pyrene stress and to establish their response profile. Three related objectives were defined for this study: (i) to assess the diversity and abundance of the bacterial community in the rhizospheres and non-rhizospheres of mangroves; (ii) to assess the distribution of different soil nutrients and their influence on bacterial diversity and abundance; and (iii) to establish the profile of the bacterial community during acclimation under pyrene stress.

## 2 Materials and methods

### 2.1 Sample collection

Samples were collected in the northeast of Shantou, Guangdong, China (longitude: 23°55′9″N, latitude: 117°25′9″E; altitude: 0 m) at the coastal zone of Yunxiao. The rhizosphere samples were collected by selecting three zones, labeled R1, R2, and R3, extending from the intertidal zone to the deeper areas of the mangrove forest. Rhizosphere sediments were defined as those directly adhering to the roots of mangrove plants, while non-rhizospheres were those collected 3 m away from the mangrove roots (Li et al., 2016). Polyvinyl chloride pipes of 4.2 cm in diameter and 50 cm in length were used to collect sediments at depths ranging from 0 to 20 cm, as described by Allaouia et al. (2022). Depths were designated as follows: Ni-1 (0–5 cm), Ni-2 (5–10 cm), Ni-3 (10–15 cm), and Ni-4 (15–20 cm), where N represents either R (rhizosphere) or NR (non-rhizosphere). After sediments were collected, basic treatments, such as removing small pebbles, stones, and roots, were performed on-site. Samples were then transported to the laboratory for biological and molecular analyses. Each sample was divided into two groups: one stored at 4 °C for determining the physical and chemical characteristics of the sediments, and the other stored at −20 °C for DNA analysis before DNA extraction.

### 2.2 Determination of physicochemical properties of sediments

Once the samples were collected, a hand-held thermometer (model no. LBT-4S, Xi’an Lonn M&E Equipment Co., Ltd.), a pH meter (Benchtop pH meter inoLab® pH 7,110 Set 4), and an oxidation–reduction potential (ORP) meter (PINPOINT® ORP Monitor) were used to measure temperature, pH, and ORP values at different depths. To determine the physical and chemical characteristics of rhizosphere and non-rhizosphere sediments, they were air-dried, crushed, and sieved through a 2-mm mesh. Next, 0.5 g of the crushed sediment sample was added to an Erlenmeyer flask and digested using the aqua regia extraction method (Victório et al., 2020). After digestion, the sample was diluted to 50 mL with deionized water and filtered through filter papers. The microelements and nutrients such as iron (Fe), manganese (Mn), zinc (Zn), magnesium (Mg), potassium (K), and calcium (Ca) were analyzed by inductively coupled plasma optical emission spectrometry (ICPOES) (Allaouia et al., 2022; Asnat et al., 2022; Elyamine et al., 2023). In contrast, other nutrients, such as total nitrogen (TN), nitrate (NO_3_-), and nitrite (NO_2_-), were determined using the Kjeldahl method as described in Konopiński (2020). A double digestion with H_2_SO_4_/HClO4 was used to analyze the phosphorus content. Carbon and sulfur were determined by dry combustion using a high-temperature induction furnace as described in Lavkulich et al. (1970).

### 2.3 Pyrene stress and succession of culture in the mineral salt medium

The method used to design MSM and to determine the effect of pyrene stress during the acclimation was inspired by Hui et al. (2018), with a slight personal modification. The details of the method and the composition of the mineral salt culture medium can be found in our previous study (Elyamine et al., 2023). Briefly, 5 g of sediment was added to 50 mL of MSM containing 50 mg/L of pyrene, and the mixture was incubated in a shaker at 25 °C and 150 rpm for 30 days. Each treatment was performed in three replicates to ensure statistical validity and scientific rigor. Every 30 days, 5 mL of the old sample was transferred to a new MSM, and genomic DNA from each old individual was extracted and stored at −80 °C for optimal preservation until further processing.

### 2.4 DNA extraction, amplification, and computational analysis

The PowerSoil® DNA isolation kit (MoBio Laboratories, Carlsbad, CA, USA) was used to extract total genomic DNA from the different samples before and after acclimation. PCR amplification of 16S rRNA genes from the V3-V4 region of the samples was performed using the same method described in our previous study (Allaouia et al., 2022; Asnat et al., 2022; Elyamine et al., 2023) using the 338F universal primers (5’-ACTCCTACGGGAGGCAGCAG-3′) and 806R (5’-GGACTACHVGGGTWTCTAAT-3′). The extracted DNA was sent to the Sangon Biotec Institute (SBI) platform in Shanghai, China, for sequencing. DNA concentrations and purity were measured using a NanoDrop 2000 spectrophotometer (Thermo Fisher Scientific, USA). Computational analysis was performed as described in Elyamine et al. (2023). Indeed, the duplication and filter-qualification of the raw fastq files, sequence classification, annotation, and alpha diversity distance calculations were performed using Quantitative Insights Into Microbial Ecology (QIIME version 1.9). UPARSE software (version 7.0.1001) was used to group the filtered sequences, with Operational Taxonomic Unit (OTU) clustered with a 97% similarity cutoff. At a 97% confidence threshold, the taxonomy of each 16S rRNA gene sequence was analyzed using the 16S rRNA database and the Ribosomal Database Project (RDP) classifier (version 2.12). The functional gene composition of the bacterial community was determined using PICRUST.

### 2.5 Estimation of pyrene degradation

To assess the extent of pyrene degradation by bacteria present in MSM, at each generation, the concentration of pyrene was extracted using dichloromethane as an extracting solvent and analyzed using high-performance liquid chromatography (HPLC) as described in previous studies (Willis et al., 1996; Wang et al., 2016a; Allaouia et al., 2022; Asnat et al., 2022). After six successive transfers, the pyrene concentration was found to be stable. Along with pyrene concentration, the microbial community in each transfer was also determined.

### 2.6 Statistical analysis

Data on sediment characteristics and pyrene degradation concentration were processed using analysis of variance (ANOVA) in SPSS software (version 20). The Duncan *post-hoc* test with a 95% confidence level was used to identify differences among the multiple stepwise means. For taxonomic analysis, similarity analysis (ANOSIM) was used to assess similarities between different experimental groups. The alpha diversity and species richness of the different communities in *in situ* and *ex situ* environments were assessed using the Chao1 and Shannon indices, calculated in the R language software (version 3.6.0) (Konopiński, 2020; Konopiński, 2020). The Shannon index was interpreted as follows: the higher the Shannon value, the higher the community diversity. At a 97% confidence threshold, the taxonomy of each 16S rRNA gene sequence was analyzed using the 16S rRNA database and the RDP classifier (version 2.12). The distance matrix and similarity or difference in sample community composition were performed using Canonical Correlation Analysis (CCA) and UniFrac distances, respectively, in QIIME version 1.9.01. The graphs for relative abundances and taxonomies were generated using the R language (version 3.6.0) and OriginPro 8. SigmaPlot 12 software was used to plot the pyrene degradation curves in the different experimental groups.

## 3 Results

### 3.1 Physical and chemical properties in the rhizosphere and non-rhizosphere

Rhizosphere and non-rhizosphere physical and chemical properties are shown in Table 1. The pH values for the rhizosphere and non-rhizosphere showed no significant difference. However, in the rhizosphere (R), the pH was slightly lower than that of the non-rhizosphere (NR).

**Table 1:** Different physicochemical properties of both rhizosphere and non-rhizosphere sediments at different depth layers.

Exp group	pH	NO_2_- (mg/L)	NO_3_- (mg/L)	NH_3_-N (mg/L)	C (%)	S (%)	Ca (mg/L)	K (mg/L)	P (mg/L)	ORP
NR	6.7	0.014 ± 0.04	1.02 ± 0.01^ab^	1.02 ± 0.07^ab^	1.90^c^	0.56^b^	15.45 ± 0.2^b^	143.03 ± 45^ab^	4.38 ± 0.7^b^	22.00 ± 8.4^d^
R1	5.22	0.03 ± 0.05	1.10 ± 0.02^ab^	0.86 ± 0.03^b^	2.16^b^	0.48^b^	16.80 ± 0.5^b^	138.7 ± 54^ab^	6.81 ± 0.5^ab^	45.83 ± 7.2^b^
R2	5.5	0.014 ± 0.05	1.14 ± 0.01^ab^	0.70 ± 0.01^b^	2.24^b^	1.53^a^	16.07 ± 0.7^b^	135.9 ± 65^ab^	6.88 ± 0.4^ab^	18.16 ± 6.2^e^
R3	5.4	0.02 ± 0.06	1.68 ± 0.03^ab^	0.61 ± 0.07^b^	5.27^ab^	1.52^a^	15.34 ± 0.4^b^	115.21 ± 51^c^	5.18 ± 0.5^b^	−18.41 ± 6.4^e^
NR1	6.6	0.01 ± 0.04	1.49 ± 0.01^ab^	0.75 ± 0.01^b^	2.34^b^	0.79^b^	12.55 ± 0.8^b^	114.85 ± 63^c^	3.56 ± 0.8^c^	56.00 ± 5.2^b^
NR2	6.65	0.03 ± 0.03	0.76 ± 0.01^b^	1.73 ± 0.08^ab^	1.45^c^	0.52^b^	13.82 ± 0.3^b^	146.51 ± 71^ab^	4.16 ± 0.5^b^	23.66 ± 7.3^d^
NR3	6.71	0.01 ± 0.06	0.76 ± 0.04^b^	2.10 ± 0.02^a^	1.94^c^	1.46^a^	14.73 ± 0.4^b^	161.23 ± 54^a^	5.02 ± 0.8^b^	37.55 ± 8.1 ^c^
NR4	6.83	0.018 ± 0.03	0.61 ± 0.06^b^	2.51 ± 0.04^a^	1.80^c^	1.44^a^	20.70 ± 0.5^a^	149.54 ± 46^ab^	4.7 ± 0.7^b^	−19.16 ± 7.4^c^
R1-1	5.77	0.08 ± 0.03	1.18 ± 0.05^ab^	0.35 ± 0.01^b^	2.13^b^	0.58^b^	17.86 ± 0.5^a^	126.44 ± 49^b^	4.99 ± 0.2^b^	84.33 ± 13.5^b^
R1-2	5.5	0.012 ± 0.05	1.80 ± 0.08^ab^	0.31 ± 0.04^b^	2.24^b^	0.92^b^	17.04 ± 0.6^b^	153.14 ± 64^ab^	8.01 ± 0.7^a^	62.66 ± 19.3^b^
R1-3	5.24	0.002 ± 0.03	1.25 ± 0.06^ab^	1.26 ± 0.02^ab^	2.20^b^	1.21^a^	16.65 ± 0.7^b^	150.15 ± 51^ab^	7.56 ± 0.8^a^	17.00 ± 9.5^e^
R1-4	5.36	0.003 ± 0.02	2.16 ± 0.07^a^	1.51 ± 0.07^ab^	2.09^b^	1.22^a^	15.64 ± 0.5^b^	125.16 ± 49^b^	6.67 ± 0.5^ab^	19.33 ± 6.5 ^e^
R2-1	5.67	0.015 ± 0.06	1.42 ± 0.05^ab^	0.98 ± 0.02^b^	2.17^b^	0.31^b^	17.73 ± 0.6^a^	131.88 ± 51^ab^	7.53 ± 0.4^a^	41.66 ± 7.3^c^
R2-2	5.6	0.01 ± 0.07	1.33 ± 0.05^ab^	0.80 ± 0.04^b^	2.30^b^	0.21^b^	16.86 ± 0.8^b^	156.46 ± 68^a^	8.25 ± 0.7^a^	51.66 ± 7.2 ^b^
R2-3	5.48	0.01 ± 0.03	1.88 ± 0.02^ab^	1.22 ± 0.08^ab^	2.09^b^	1.27^a^	15.45 ± 0.4^b^	133.14 ± 67^ab^	6.40 ± 0.3^ab^	40.66 ± 9.6^c^
R2-4	5.28	0.002 ± 0.04	2.92 ± 0.06^a^	1.22 ± 0.04^ab^	2.41^b^	1.34^a^	14.23 ± 0.6^b^	122.33 ± 62^b^	5.33 ± 0.2^b^	−101.33 ± 21.6^a^
R3-1	5.67	0.01 ± 0.06	1.67 ± 0.06^ab^	0.63 ± 0.06^b^	2.04^b^	0.61^b^	14.04 ± 0.7^b^	111.91 ± 43^c^	5.55 ± 0.7^b^	45.33 ± 8.3^c^
R3-2	5.17	0.07 ± 0.05	1.96 ± 0.03^ab^	0.59 ± 0.01^b^	2.44^b^	0.78^b^	15.67 ± 0.5^b^	113.28 ± 64^c^	5.08 ± 0.3^b^	33.66 ± 6.4^c^
R3-3	5.42	0.01 ± 0.04	2.72 ± 0.02^a^	0.92 ± 0.03^b^	7.93^a^	1.35^a^	15.01 ± 0.7^b^	116.22 ± 52^c^	4.83 ± 0.6^b^	−61.00 ± 8.2^b^
R3-4	5.45	0.004 ± 0.03	2.37 ± 0.04^a^	0.82 ± 0.02^b^	8.65^a^	1.34^a^	16.66 ± 0.8^b^	119.43 ± 45^c^	5.27 ± 0.6^b^	−51.66 ± 7.5^b^

Data are the mean of three replicates ± SD and were compared by Duncan’s multiple range test. Lowercase letters indicate significant differences at *p* < 0.05.

#### 3.1.1 Nitrate and nitrite concentration

Nitrogen forms were found to vary from the rhizosphere to the non-rhizosphere and from one depth to another. Nitrate concentration (NO_3_-) was significantly higher in the rhizosphere than in the non-rhizosphere. When the non-rhizospheres were compared across the different layers, the concentration in the surface layer (NR1) was higher than in the underlying layers (NR2-3 and NR2-4). This indicated that in the non-rhizosphere, the greater the depth, the lower the nitrate concentration. Contrary to the non-rhizosphere, in the deeper sampling compartments of the rhizosphere (R1-4, R2-4, and R3-4), the nitrate concentration was significantly higher (*p* < 0.01). The nitrite concentration (NO_2_-) in both rhizosphere and non-rhizosphere samples showed no significant difference. Thus, we can speculate that the enrichment of nitrogen in the non-rhizosphere may be attributed to the process of net mineralization. Indeed, in the vicinity of plants, namely in the rhizosphere, plants excrete root-derived exudates, which not only can contribute to mineralization but also can be immobilized within organic matter, thereby modifying the redox conditions in the rhizosphere.

#### 3.1.2 Ammonia nitrogen, carbon, and sulfur concentration

Although the concentration of ammonia nitrogen (NH_3_-N) was generally higher in the non-rhizosphere than in the rhizosphere, in NR1-3 and NR1-4, the concentration was higher than in other experimental group layers. However, in both non-rhizosphere and rhizosphere, and particularly in the non-rhizosphere samples NR3 and NR4, the greater the depth, the higher the NH_3_-N concentration.

Carbon concentration in R3-3 and R3-4 was significantly higher than in the other experimental groups. Furthermore, the concentration in the non-rhizosphere samples, including the different sampling depths, was significantly (*p* < 0.05) lower than in the rhizosphere sampling compartments. Sulfur concentration was higher in the underlying compartments (10–20 cm) than in the top layer. However, no significant difference was observed between the rhizosphere and the non-rhizosphere.

#### 3.1.3 Calcium, potassium, phosphorus concentration and ORP

Calcium concentration was significantly (*p* < 0.05) higher in the non-rhizosphere at 15–20 cm depth (NR4) and in the rhizosphere (R1 and R2) at the surface (0–5 cm). No significant difference was noted between the rhizosphere and non-rhizosphere samples for potassium concentration (K). However, taking into account the different depth layers, the concentration in the non-rhizosphere at 10–15 cm (NR3) and the rhizosphere at 5–10 cm (R2-2) was significantly higher (*p* < 0.001) than in the other experimental groups. The lowest concentration was observed in rhizosphere R3 at all depth layers and in the non-rhizosphere NR1 at the surface layer 0–5 cm. Phosphorus concentration was significantly higher in the rhizosphere at the surface layer (R2-1, R1-2, and R1-3), while the lowest concentrations were observed in non-rhizosphere samples. The ORP in the deep zone (15–20 cm) of both rhizosphere and non-rhizosphere was found to be negative, and positive at the upper layer, indicating, respectively, reduction and oxidation processes. Indeed, it was found that oxidation occurs in the surface area, while reduction occurs in the deeper compartments. This result was consistent with previous research, which showed that in mangrove sediments, the oxidation–reduction potential is highly variable (Wang et al., 2016b).

#### 3.1.4 Microelements concentration

Micronutrients that play an important role in soil microbial life, such as zinc (Zn), iron (Fe), manganese (Mn), and magnesium (Mg), were measured and presented in Table 2. Considering the different experimental groups and also the “depth” factor, the distribution of Zn was almost similar and showed no significant difference. However, the lowest concentrations were measured in the rhizosphere samples R2-4, R3-1, and R3-2 and in the upper level of the non-rhizosphere (NR1). The Fe concentration in the non-rhizosphere (NR1 and NR2) was notably lower, while in the rhizosphere (R1-3 and R2-2), it was significantly higher than in the other experimental groups. The Mn concentration in the different experimental groups showed a clear difference between the rhizosphere and the non-rhizosphere. In the rhizosphere (R1-1 and R2-1), Mn was higher, while in the non-rhizosphere (NR2), it was lower. The concentration of Mg measured in both rhizosphere and non-rhizosphere showed no significant difference.

**Table 2:** Different microelements, bacterial OTUs, and estimated bacterial abundance and diversity alpha indices in both rhizosphere and non-rhizosphere sediments at different depth layers.

Exp group	Fe (mg/L)	Mg (mg/L)	Mn (mg/L)	Zn (mg/L)	Seq number	Total OTUs	Shannon index	Chao1 index
NR	10.61 ± 0.5^⁎^	4.11 ± 0.5	0.25 ± 0.02^ab^	0.23 ± 0.02^a^	55,494	121,774^ab^	3.21 ± 0.52^ab‡^	730.63 ± 34^b‡^
R1	16.47 ± 0.6^⁎⁎^	5.01 ± 0.6	0.22 ± 0.04^ab^	0.22 ± 0.04^a^	60,226	121,521^ab^	3.96 ± 0.61^ab‡^	776.72 ± 28^ab‡‡^
R2	14.72 ± 0.8^⁎⁎^	5.09 ± 0.5	0.21 ± 0.02^ab^	0.19 ± 0.03^ab^	58,870	122,158^ab^	4.08 ± 0.69^a‡‡^	749.36 ± 41^ab‡‡^
R3	11.26 ± 0.5^⁎^	4.62 ± 0.8	0.17 ± 0.02^b^	0.15 ± 0.04^b^	59,088	121,198^ab^	4.14 ± 0.37^a‡‡^	742.41 ± 47^ab‡‡^
NR1	7.87 ± 0.6^⁎^	3.38 ± 0.2	0.18 ± 0.05^b^	0.23 ± 0.05^a^	57,475	125,928^††† b^	3.71 ± 0.44^ab††^	724.15 ± 42^ab†††^
NR2	9.02 ± 0.7^⁎^	3.70 ± 0.3	0.14 ± 0.04	0.24 ± 0.04^a^	56,772	124,296^††† b^	3.69 ± 0.25^ab††^	712.22 ± 51^ab††^
NR3	13.27 ± 0.5^⁎⁎^	4.71 ± 0.5	0.24 ± 0.04^ab^	0.24 ± 0.04^a^	53,937	122,602^†† ab^	2.96 ± 0.15^b†^	495.05 ± 39^c†^
NR4	12.29 ± 0.6^⁎⁎^	4.67 ± 0.6	0.24 ± 0.05^ab^	0.24 ± 0.07^a^	53,793	114,271^†† d^	2.46 ± 0.25^b†^	491.09 ± 35^c†^
R1-1	13.68 ± 0.7^⁎⁎^	4.48 ± 0.4	0.28 ± 0.03^a^	0.24 ± 0.03^a^	62,443	127,701^*** a^	4.91 ± 0.48^a**^	1,057 ± 63^a***^
R1-2	17.68 ± 0.8^⁂^	5.44 ± 0.5	0.23 ± 0.07	0.23 ± 0.06^a^	62,162	121,902^*** ab^	4.59 ± 0.37^a**^	1035.29 ± 51^a***^
R1-3	18.78 ± 0.5^⁂^	5.52 ± 0.7	0.20 ± 0.06^ab^	0.21 ± 0.06^a^	60,934	120,342^*ab^	3.1 ± 0.27^ab*^	534.42 ± 51^b**^
R1-4	15.75 ± 0.5^⁎⁎^	4.58 ± 0.4	0.18 ± 0.04^b^	0.22 ± 0.03^a^	55,365	116,140^*c^	3.23 ± 0.35^ab*^	480.17 ± 42^c*^
R2-1	12.90 ± 0.7^⁎⁎^	4.64 ± 0.4	0.27 ± 0.05^a^	0.22 ± 0.06^a^	61,273	127,271^⁑⁑⁑ a^	4.96 ± 0.38^a⁑⁑^	1045.58 ± 51^a⁑⁑⁑^
R2-2	19.09 ± 0.6^⁂^	5.80 ± 0.6	0.24 ± 0.05^ab^	0.23 ± 0.06^a^	61,142	120,111^⁑⁑ ab^	4.78 ± 0.29^a⁑⁑^	1033.62 ± 65^a⁑⁑⁑^
R2-3	12.71 ± 0.9^⁎⁎^	4.92 ± 0.3	0.18 ± 0.04^b^	0.18 ± 0.06^ab^	56,319	123,073^⁑⁑ ab^	3.34 ± 0.54^ab⁑^	505.35 ± 41^b⁑⁑^
R2-4	14.17 ± 0.8^⁎⁎^	5.02 ± 0.4	0.18 ± 0.03^b^	0.14 ± 0.04^b^	56,747	118,178^⁑ c^	3.25 ± 0.48^ab⁑^	412.88 ± 54^c⁑^
R3-1	10.94 ± 0.8^⁎^	4.43 ± 0.3	0.21 ± 0.02^ab^	0.13 ± 0.04^b^	59,124	125,048^⁂ b^	4.46 ± 0.36^a⁂^	937.59 ± 52^a⁂^
R3-2	9.81 ± 0.7^⁎^	4.34 ± 0.5	0.16 ± 0.04^b^	0.14 ± 0.03^b^	59,846	123,956^⁂ b^	4.39 ± 0.46^a⁂^	915.18 ± 49^a⁂^
R3-3	10.23 ± 0.8^⁎^	4.61 ± 0.8	0.15 ± 0.04^b^	0.16 ± 0.06^ab^	58,899	120,138^⁎⁎ ab^	4.02 ± 0.37^a⁂^	562.78 ± 42^b⁎⁎^
R3-4	14.08 ± 0.5^⁎⁎^	5.09 ± 0.4	0.17 ± 0.04^b^	0.18 ± 0.04^ab^	58,485	115,649^⁎⁎ c^	3.67 ± 0.38^ab⁎⁎^	554.07 ± 38^b⁎⁎^

Data are the mean of three replicates ± SD and were compared by Duncan’s multiple range test. Seq number refers to the quality-filtered number of sample reads. Total OTUs are the 16S rRNA sequence-based OTUs obtained by sample clustering and normalization. For microelements, different symbols *, **, and ⁂ indicate significant differences at *p* < 0.05, *p* < 0.01, and *p* < 0.001, respectively. For bacterial OTUs and diversity indexes, the symbols *, ⁕, ‡, †, ⁑, and lowercase letters (four values for each experimental group) indicate significant differences at *p* < 0.05.

### 3.2 Distribution of the bacterial community and its abundance

In total, 121,774, 121,521, 122,158, and 121,198 quality-filtered sequences were identified, respectively, in non-rhizosphere (NR) and rhizosphere samples R1, R2, and R3 through 16S rRNA gene sequencing (Table 2). No significant difference was noted in the number of identified OTUs between NR and R samples (ANOVA, *p* < 0.05). However, when the samples collected in the different rhizospheres are compared, the deeper we move into the mangrove forest, the higher the bacterial abundance becomes. Moreover, when considering “depth” as a factor, in both non-rhizosphere and rhizosphere samples, the greater the depth, the fewer OTUs were identified. This result indicated that bacterial abundance depends on the canopy, which is influenced by light availability and the depth factor. The Shannon and Chao1 indices, which were used to estimate the specific richness, showed that bacterial diversity and abundance were mostly and significantly higher in the top layer (0–10) than in the underlying samples (Table 2). No significant difference was noted in the specific richness between rhizosphere and non-rhizosphere samples.

### 3.3 Relationship between *in situ* microflora and environmental properties

**Figure 1:**
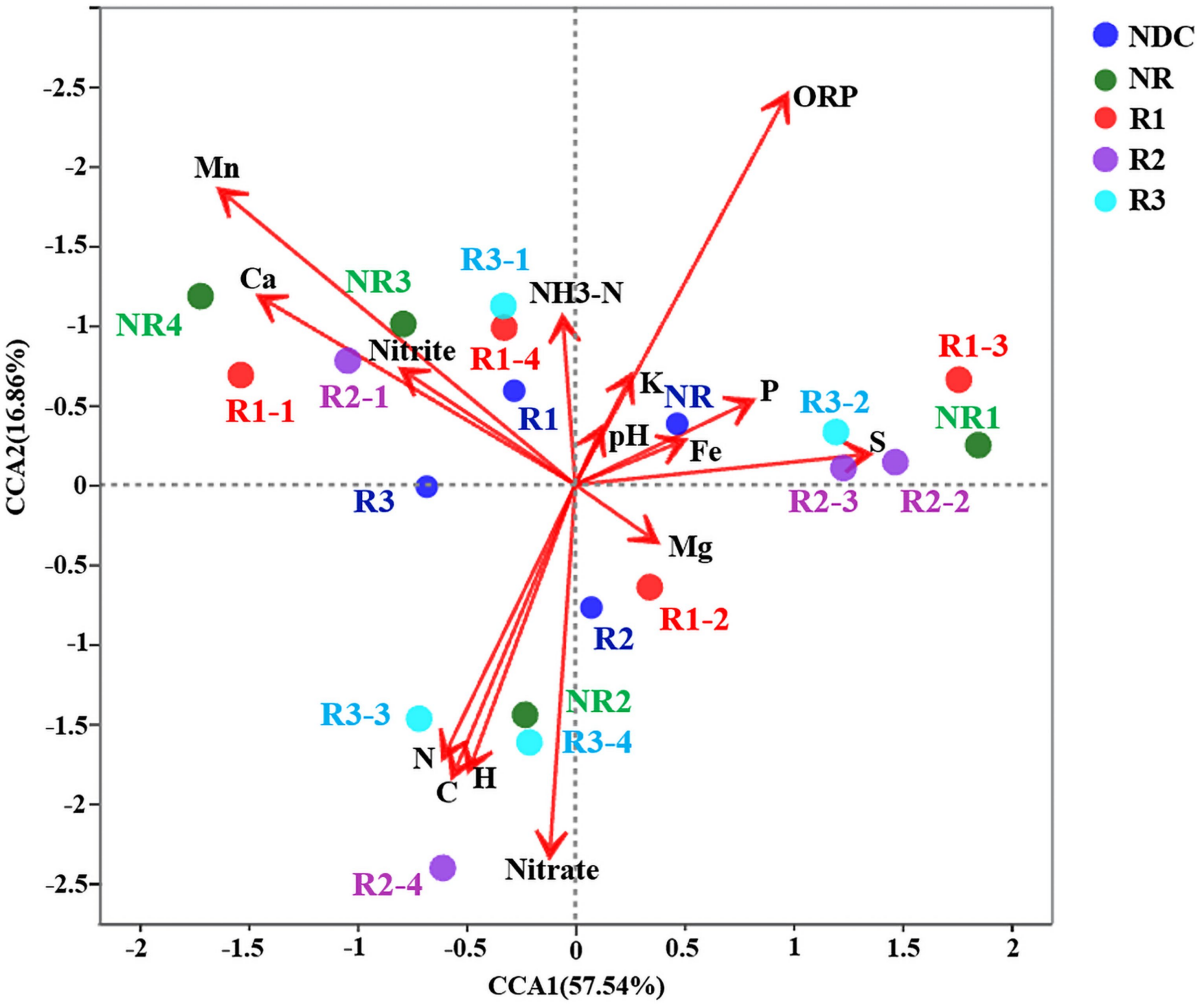
CCA analysis of bacterial abundance based on physicochemical parameters and species-level data.

The second-generation sequencing technology was used to analyze the mangrove rhizosphere and non-rhizosphere microflora *in situ* based on OTUs annotation results. In order to explore the influence of *in situ* environmental properties on the flora in the mangrove sediments, CCA analysis was performed based on 16S rRNA gene high-throughput sequencing (Figure 1).

The impact of ORP was not as important as that of K, P, pH, and Fe on the microflora of NR, R3-2, and R2-3. Ammonia nitrogen (NH_3_-N) was strongly correlated with the microflora present in the rhizosphere (R1, R1-4, and R3-1). Mn, NO_2_-, and Ca were positively correlated with the microflora in non-rhizosphere (NR3 and NR4) and rhizosphere (R1-1 and R2-1). Carbon, nitrogen, and sulfur were correlated with the microflora present in R3-3 and R2-4. Nitrate (NO3-) was strongly correlated with the microflora in R2, R3-4, and NR2. The microelement Mg was slightly correlated with the microflora in R1-2. Consequently, even though each element impacted the different experimental depth layers differently, it was revealed that the abundance of both rhizosphere and non-rhizosphere microflora clearly depends on environmental and local abiotic factors.

### 3.4 Rhizosphere and non-rhizosphere bacterial composition

**Figure 2:**
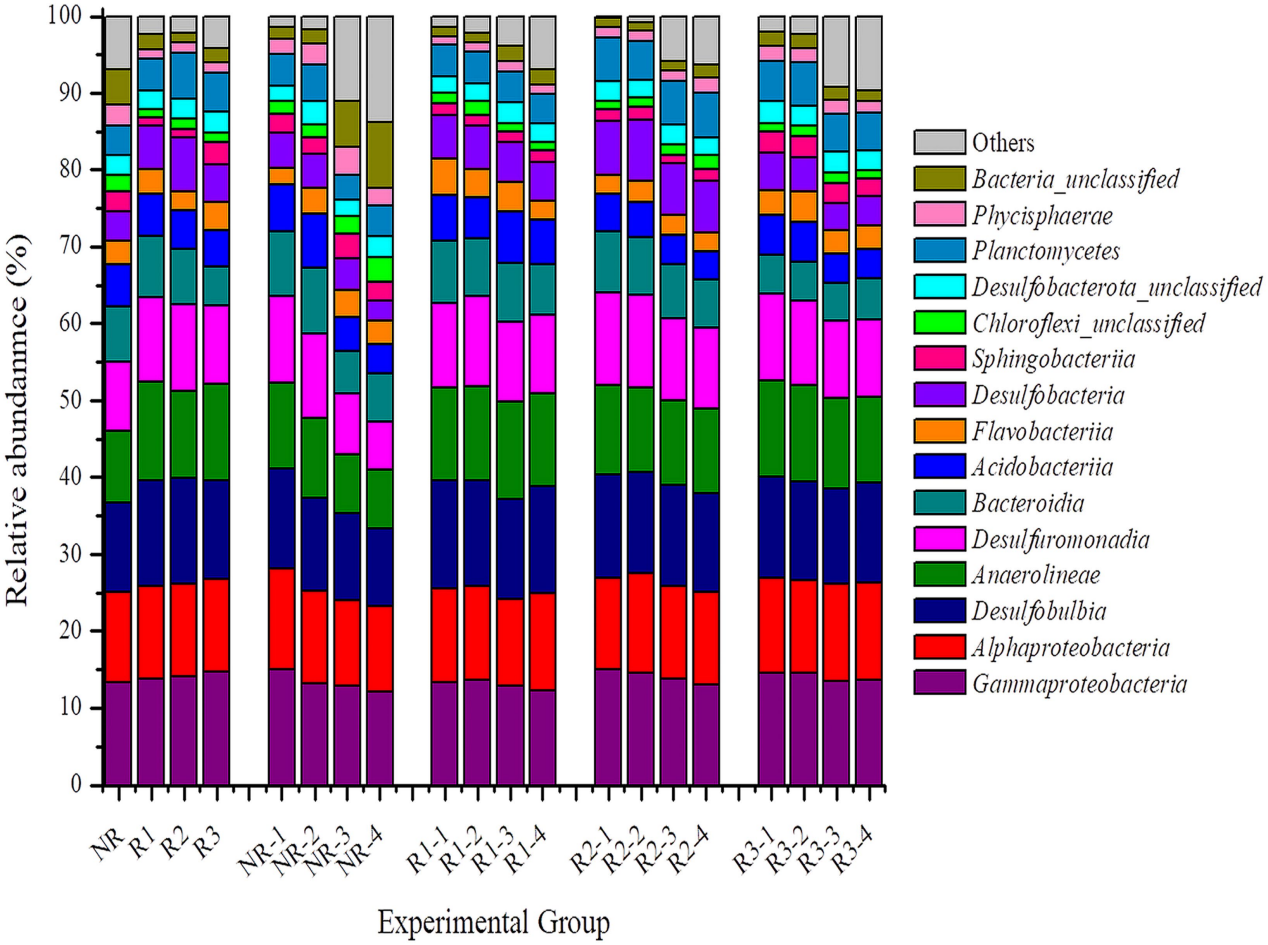
Bacterial relative abundance at the class level. The horizontal and vertical axes, respectively, represent the name of each sample and the abundance ratio in three replicates. Each color corresponds to a class name, and the different color widths indicate different abundance. NR, non-rhizosphere; R, rhizosphere.

The single-sample multi-level species composition diagram showed the mangrove rhizosphere/non-rhizosphere single-sample results, moving from the inside to the outside through multiple concentric circles (Supplementary Figure S1). From the single-sample results, it can be observed that the distribution and richness of bacteria in the mangrove ecosystem vary. However, naturally, the distribution of the bacterial community in the rhizosphere and the non-rhizosphere seems to be different. This is why the diversity and relative abundance at the phylum and class levels were evaluated and plotted in Supplementary Figure S2 and Figure 2, respectively. At the class level (Figure 2), generally, the bacterial diversity in both rhizosphere and non-rhizosphere did not show major differences. The distribution of taxonomic classes in the two experimental groups and across the different depth levels was almost the same. However, a slight difference was noted in the relative abundance between the experimental groups. To be more focused and objective, only results where the percentage was greater than 1% were considered, and those ≤1% were classified as other. Except Sphingobacteriia (2.54% vs. 1.06%), uncultured Chloroflexi (2.21% vs. 1.26%), Bacteroidia (4% vs. 1.64%), and Phycisphaerae (2.71% vs. 1.3%), which were slightly (*p* > 0.05) more abundant in the non-rhizosphere samples, all other classes were slightly more abundant in the rhizosphere samples (*p* > 0.05). The most abundant identified classes were Desulfuromonadia, Anaeroline, Desulfobulbia, Alphaproteobacteria, and Gammaproteobacteria, with 10.81, 12.57, 13.4, 12 and 14.18%, respectively, in rhizosphere sediments, compared to 9.10 9.18 11.63, 11.86, and 13.28% in the non-rhizosphere. In addition, when the “depth factor” was considered, the deeper the layer, the lower the bacterial abundance. Clearly, in the top layer (0–10 cm), microbial abundance was significantly higher, whereas at greater depths, only bacteria with a relative abundance ≤1% increased. This result supports existing evidence that, in soil, bacteria are more abundant at the surface than in the deeper compartments.

Bacterial relative abundance in both mangrove rhizosphere and non-rhizosphere was further evaluated at the phylum level (Supplementary Figure S2). The relative abundance of Planctomycetota was higher in the non-rhizosphere samples than in the rhizosphere, with 10.74 and 6.42%, respectively. Proteobacteria were the most dominant phylum, with 48.84% in the rhizosphere and 44.77% in the non-rhizosphere, followed by Chloroflexi, Bacteroidota, and Desulfobacterota, with 12.67% vs. 10, 11.88% vs. 9.79, and 8% vs. 5%, respectively. Taking the depth factor into account, in the non-rhizosphere, the upper layer (0–5 cm) harbored more bacteria than the deeper zone (10–20 cm).

### 3.5 Pyrene concentration and the pyrene-tolerating bacterial community

The mangrove rhizosphere and non-rhizosphere samples were used in an acclimation process using pyrene as an external stress factor and a carbon source, through a succession of culture incubation for 30 days in MSM. At the end of the process, the concentration of pyrene and the bacterial community present were evaluated. For the process of acclimation, since the upper layer (0–10 cm) is the most frequented by soil microbes and also since the degradation process was conducted in aerobic conditions, we only considered the results of the two top layers (0–5 and 5–10 cm).

#### 3.5.1 Pyrene concentration and progression

**Figure 3:**
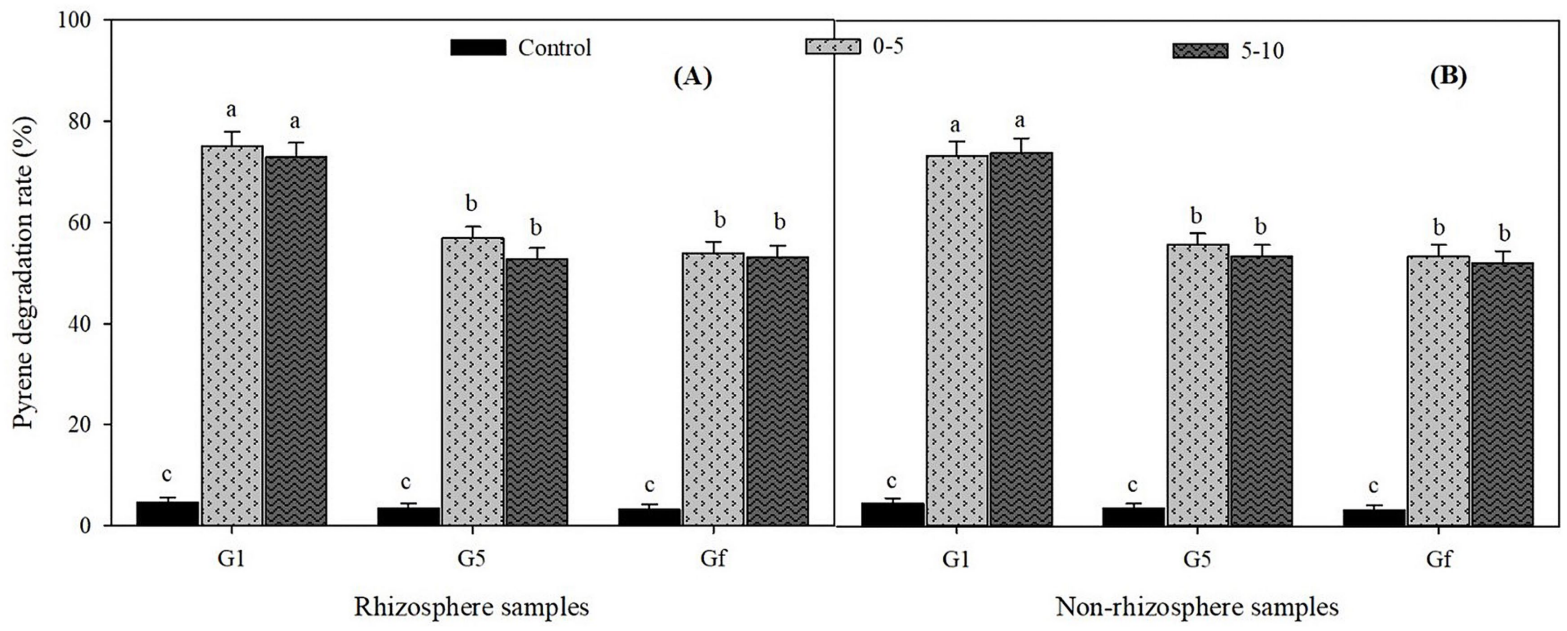
Percentage of pyrene degraded in three different generations [first (G1), before the last (G5), and the last one (Gf)] during different successions of culture for samples collected in the rhizosphere **(A)** and non-rhizosphere **(B)**. The horizontal and vertical axes, respectively, represent the generation number and the pyrene degradation rate (%) in three replicates. Different lowercase letters indicate significant differences at *p* < 0.05.

The pyrene concentration in different acclimation samples at three different succession transfers in MSM (first, before the last, and the last transfer, denoted G1, G5, and Gf, respectively) is plotted in Figure 3. The degradation rate in the control was significantly lower (*p* < 0.001) than in the experimental group. This indicates that microorganisms present in the rhizosphere and non-rhizosphere were responsible for the pyrene removal in the experimental group. In both rhizosphere (Figure 3A) and non-rhizosphere (Figure 3B) samples, the pyrene degradation rate in the first transfer (G1) was significantly (*p* < 0.05) higher than in the before the last and the last transfers (G5 and Gf). This could be explained by the enrichment of degrading microorganisms present in the first generation. The degradation rate recorded in the first transfer (G1) was approximately 75 and 72.9% for the samples collected in R1 and R2, respectively, compared to 73.2 and 72.9% recorded in NR1 and NR2 samples. Although the degradation rate recorded in the surface layer (0–5 cm) seems to be slightly higher than that of the middle layer (5–10 cm), no significant difference was noted in rhizosphere and non-rhizosphere samples. In G5 and Gf, the degradation rate was similar, with 53 and 51% at 0–5 cm and 5–10 cm layers, respectively. This indicated that the microbial community present in the culture was stable.

### 3.6 Bacterial changes and the pyrene-tolerant bacterial community

**Figure 4:**
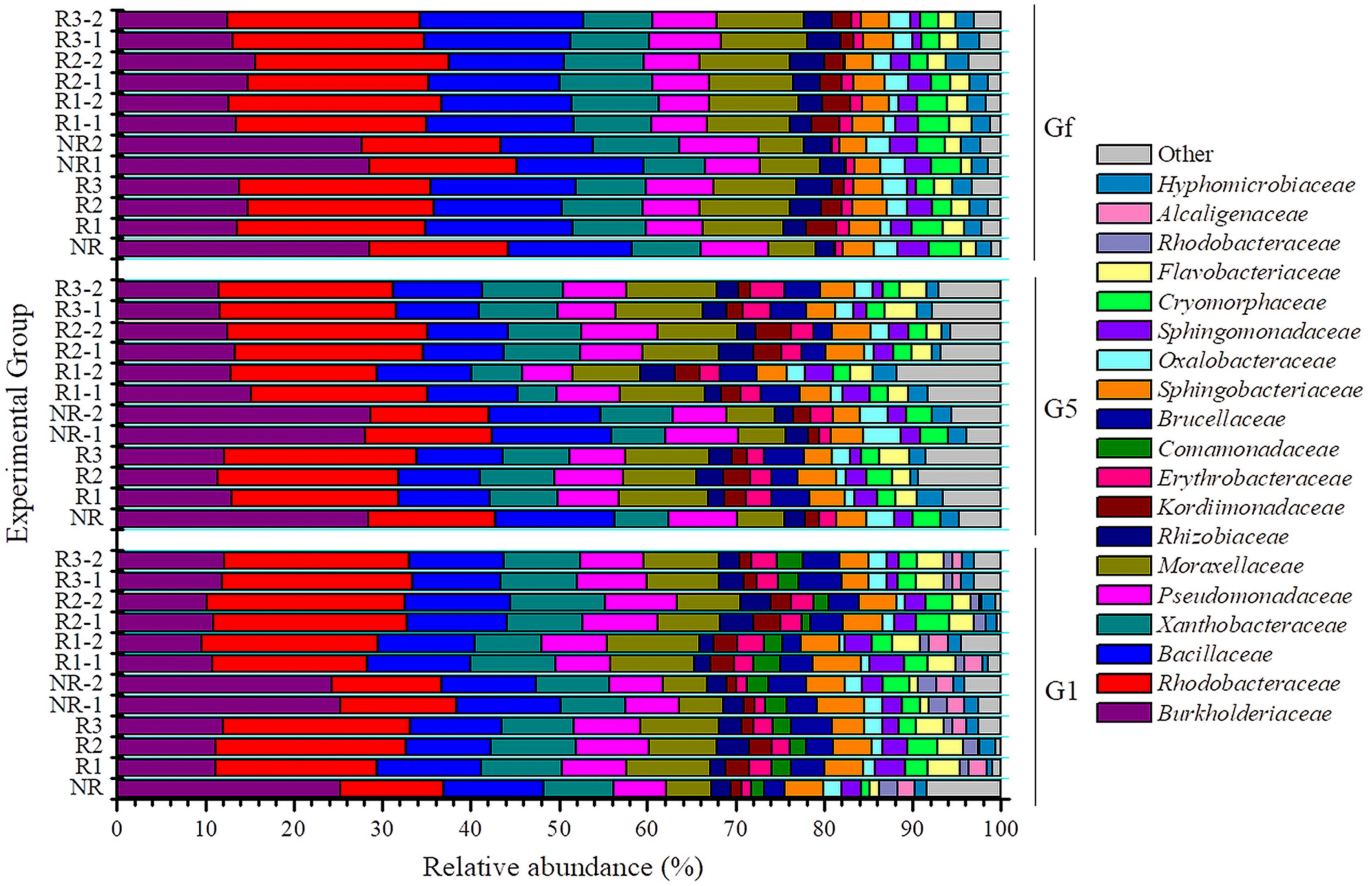
Bacterial relative abundance at the family level determined in three different generations (first (G1), before the last (G5), and the last one (Gf)) during different successions of culture for the sample. The horizontal and vertical axes, respectively, represent the name of each sample and the abundance ratio in three replicates. Each color corresponds to a family name, and the different color widths indicate different abundance. NR, non-rhizosphere; R, rhizosphere.

**Figure 5:**
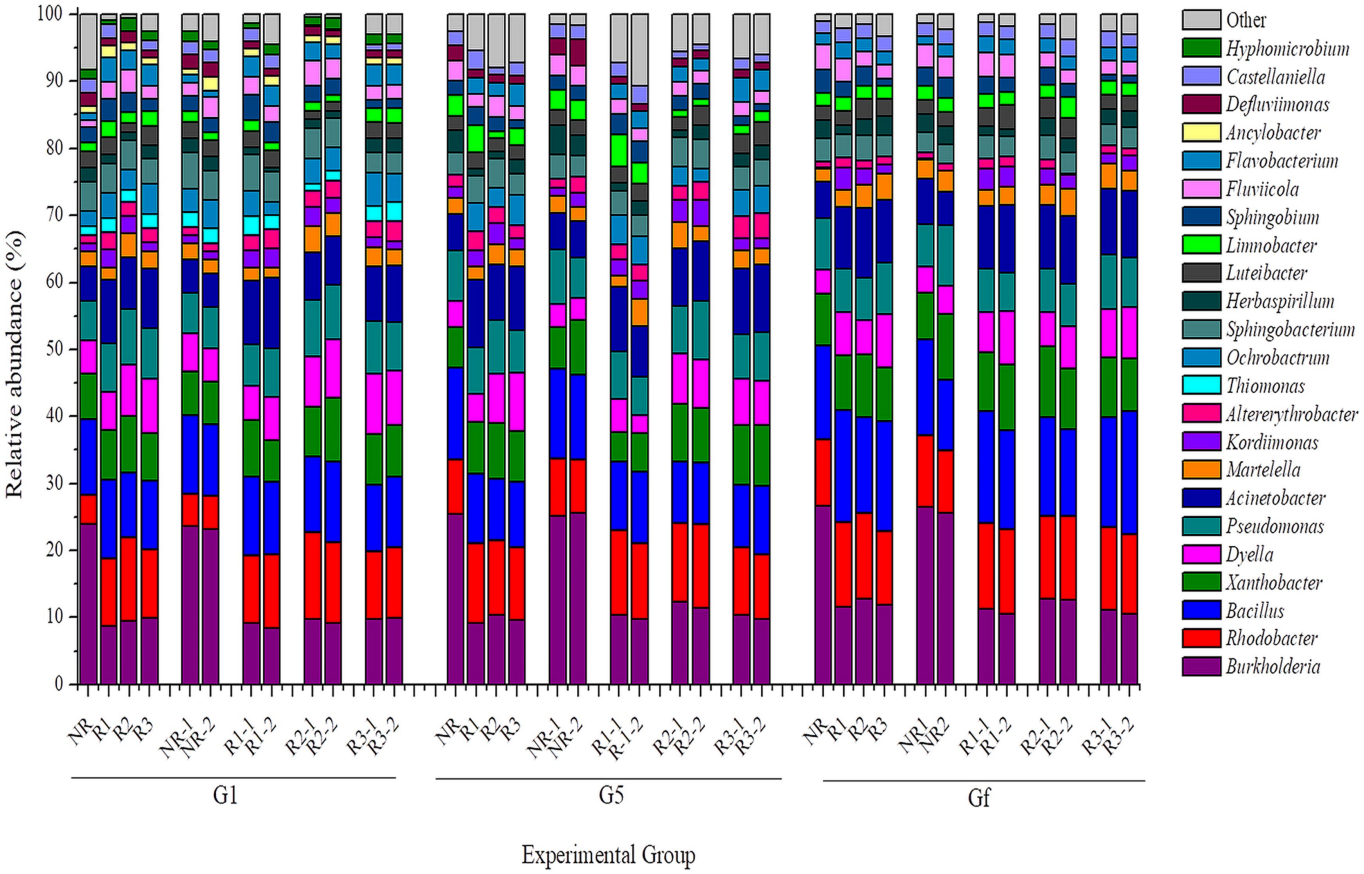
Bacterial relative abundance at the genus level determined in three different generations [first (G1), before the last (G5), and the last one (Gf)] during different successions of culture for the sample. The horizontal and vertical axes, respectively, represent the name of each sample and the abundance ratio in three replicates. Each color corresponds to a genus name, and the different color widths indicate different abundance. NR, non-rhizosphere; R, rhizosphere.

To assess the different bacterial communities after the different cultural transfers (first, before the last, and the last transfer, denoted G1, G5, and Gf, respectively) under pyrene stress, Illumina pairwise sequencing of the 16S rRNA gene was performed. The analysis of bacterial abundance indicated that the *in situ* community had the greatest abundance among the acclimated communities. Indeed, a significant difference was noted when we compared bacterial community abundance during the acclimation period with that *in situ*; this is probably due to the pollutant stress in both the rhizosphere and the non-rhizosphere. The bacterial communities in mangrove rhizosphere and non-rhizosphere sediments after acclimation were classified at different taxonomic levels, including class (Supplementary Figure S3), family (Figure 4), and genus levels (Figure 5). As in the *in situ* samples, OTUs whose relative abundance was ≤1% were classified as other. In the first transfer (G1), the most identified OTUs were divided into 19 families (Figure 4), dominated by Burkholderiaceae (25% vs. 11.43% in non-rhizosphere vs. rhizosphere), Rhodobacteraceae (11% vs. 20%), Bacillaceae (11% vs. 10.59%), Xanthobacteraceae (7.84% vs. 8.97%), Pseudomonadaceae (6% vs. 7.70%), and Moraxellaceae (5% vs. 8.63%). However, in both before the last and the last transfers (G5 and Gf), these dominant families increased slightly. A few OTUs belonging to Burkholderiaceae, Rhodobacteraceae, and Bacillaceae increased in G5, with 28.37% vs. 12, 14.36% vs. 20 and 13% vs. 9.77% in non-rhizosphere vs. rhizosphere samples, respectively. In Gf, these families increased to 28.59% vs. 14, 15.67% vs. 21.36, and 13.96% vs. 15.84%, respectively, in non-rhizosphere vs. rhizosphere samples.

The bacterial classification at the genus level (Figure 5) also revealed clear changes. In total, 23 genera were identified in the first transfer (G1), dominated by *Burkholderia* (23.9% vs. 9.23%), *Rhodobacter* (4% vs. 10.95%), *Bacillus* (11% vs. 10%), *Xanthobacter* (6.82% vs. 7.62%), *Dyella* (4.9% vs. 7%), *Pseudomonas* (6% vs. 7.70%), and *Acinetobacter* (5% vs. 8.63%), respectively, in non-rhizosphere vs. rhizosphere samples. In the other two transfers (G5 and Gf), Thiomonas, Ochrobactrum, Ancylobacter, Defluviimonas, and Hyphomicrobium were almost absent, whereas the dominant genera increased slightly. This was the case for *Burkholderia*, *Rhodobacter*, *Bacillus*, and *Pseudomonas*, which accounted for approximately 25% vs. 9.65, 8% vs. 11, 13% vs. 9.77, and 7% vs. 8% in G5 and 26% vs. 12, 9% vs. 12, 13% vs. 15, and 7% vs. 8% in Gf.

### 3.7 Correlation between bacterial communities in different samples

The graphic in Supplementary Figure S4 shows the heatmap correlation matrix representing the correlation between different OTUs identified in the rhizosphere and non-rhizosphere samples. This correlation helps to understand the linear relationship between the different identified OTUs in the different experimental groups. OTUs identified in the rhizosphere sample R2-2 were positively correlated with those of the non-rhizosphere sample NR2 (r = 0.93, *p* = 0.02), the rhizosphere samples R3-2 (r = 0.72, *p* = 0.03) and R1-1 (r = 0.81, *p* = 0.03), the non-rhizosphere sample NR1 (r = 0.74, *p* = 0.04), and the rhizosphere samples R1-2 (r = 0.69, *p* = 0.03) and R3-1 (r = 0.62, *p* = 0.03). Interestingly, the identified OTUs in the non-rhizosphere sample NR1 were highly correlated with the non-rhizosphere sample NR2 (r = 0.96, *p* = 0.04).

The scatterplot matrix presented in Supplementary Figure S5 highlights the correlation between different genera identified in the experimental groups during the acclimation process under pyrene stress. The genus *Bacillus* was strongly correlated with *Pseudomonas* and *Martelella* (r = 0.83, *p* = 0.04). Similarly, *Xanthobacter* was correlated with *Pseudomonas*, *Martelella, Altererythrobacter*, and *Sphingobium* (r = 0.86, *p* = 0.02). The genus *Dyella* was correlated with *Martelella, Altererythrobacter*, and *Sphingobium* (r = 0.88, *p* = 0.03). *Altererythrobacter* was positively correlated with *Pseudomonas* and *Sphingobium* (r = 0.81, *p* = 0.02), and finally, the genus *Sphingobacterium* was correlated with *Altererythrobacter* and *Kordiimonas* (r = 0.77, *p* = 0.04).

### 3.8 Core genome under pyrene stress

**Figure 6:**
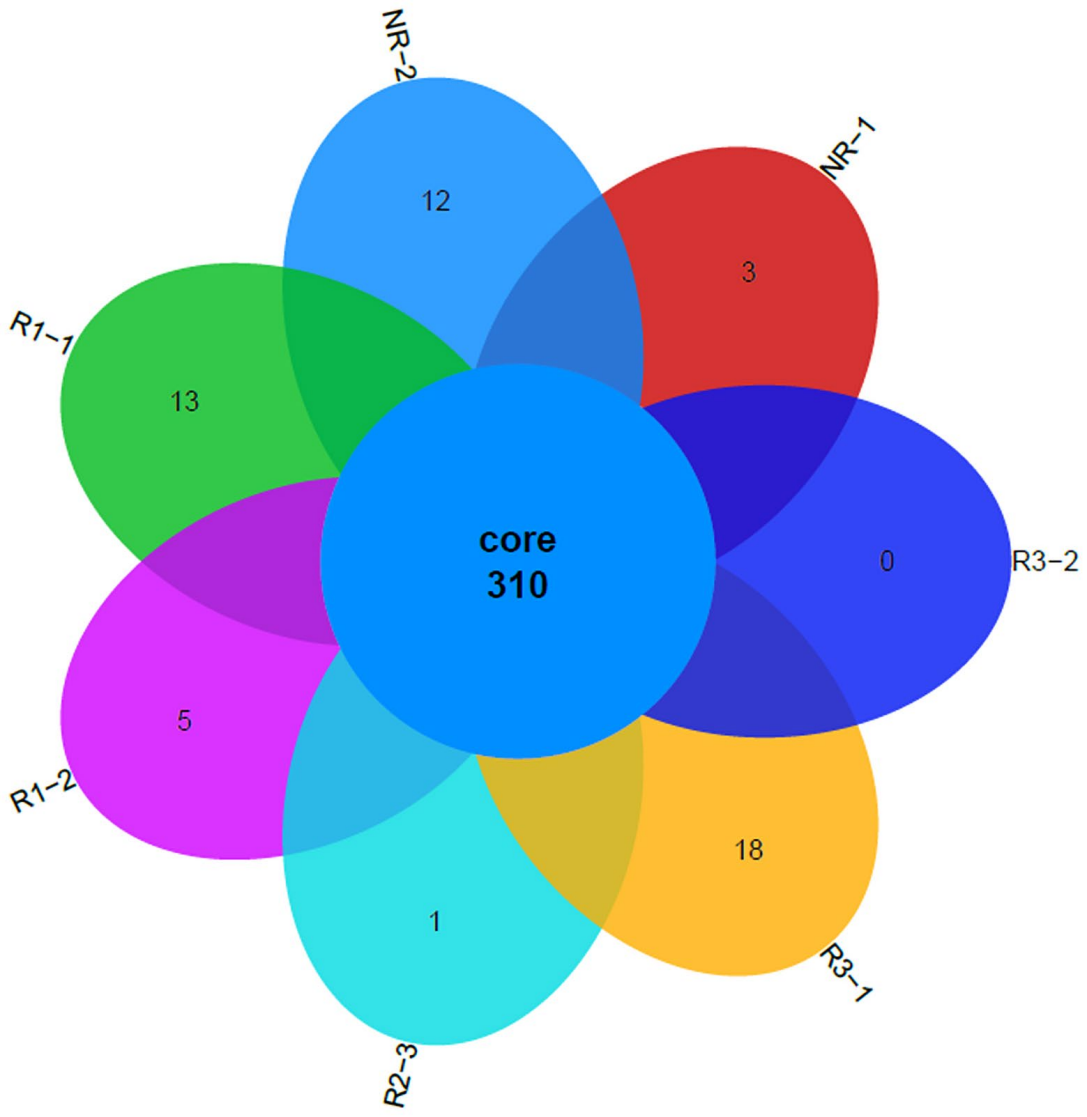
Core microbiome OTUs identified after the acclimation process of rhizosphere and non-rhizosphere samples. Each color corresponds to an experimental group name, and the number on each color corresponds to the abundance of the identified core OTUs. NR, non-rhizosphere; R, rhizosphere.

The bacterial community structure of the different experimental groups during the acclimation process under pyrene stress was similar in the top layer. Therefore, it was crucial to identify and determine common bacteria among the different experimental groups. A core microbiome, conventionally considered as taxa present in all samples, was thereafter identified. In Figure 6, the identified core microbiome after the acclimation process of rhizosphere and non-rhizosphere samples is shown. In total, 310 OTUs, defined as the “core microbiome,” were identified, and few or no OTUs were specific to individual experimental groups. It is important to note that when we talk about genomes, we are referring to “genes,” which are putative protein-coding sequences. As we are talking about the acclimation process under pyrene stress, it can be speculated that these 310 core OTUs may have roles in tolerance to, or in the metabolism of, pyrene.

## 4 Discussion

### 4.1 Influence of physical and chemical properties on the R and NR bacterial community

The variation of pH in this study is consistent with numerous scientific reports and studies. Numerous chemical reactions in soil and sediment are linked to the pH value. Indeed, it is reported by several studies that nutrient availability in general, and its accessible form, depends largely on the soil pH (Schneider et al., 2013; Tripathi et al., 2019). The soil pH is known to be influenced by various factors in soil and sediment, especially root respiration and soil microorganisms, which are a source of proton (H+) production, thus lowering the pH in soil and sediment (Hinsinger et al., 2003), and in the present case, the rhizosphere compartment. This can explain why a slightly lower pH was observed in the rhizosphere than in the non-rhizosphere. Nitrogen forms were found to vary from the rhizosphere to the non-rhizosphere and from one depth to another. This result is consistent with the existing knowledge on nutrients in rhizosphere and non-rhizosphere sediment. Indeed, mineralization is one of the main processes by which soil sediment enriches itself in different nutrients (Vicente et al., 2018). In the rhizosphere, both mineralization and immobilization of different nutrients by sediments can occur simultaneously and reach equilibrium (Liu et al., 2020). This could explain the difference found in different nitrogen forms in one compartment and the other.

The concentration of ammonia nitrogen (NH_3_-N) was generally higher in the non-rhizosphere than in the rhizosphere. This observed variation could be explained by rhizosphere priming. Indeed, research has shown that priming, defined as the short-term change in the mineralization of organic matter originating in the soil due to the release of C deposited by fresh roots *via* root exudates, cells sloughing, and epidermal cell death, can influence soil N cycling (Zhang et al., 2013; Pathan et al., 2015; Wanapaisan et al., 2018)). On one hand, this system is considered a kind of evolutionary mutualism between plants and rhizosphere microbes that benefit from plant-derived C and, in turn, mutually benefit plants through enhanced microbial mineralization of organic N from the soil. On the other hand, studies demonstrate that the effects of priming on soil N cycling and stimulation of extracellular enzymes involved in N mineralization are a key mechanism in mobilizing soil nutrients (Dash et al., 2013; Díaz et al., 2013). It is clear that the effects of priming on soil nitrogen cycling can vary considerably depending on many factors such as the identity and phenology of plant species, microbial status, and soil type (Hinsinger et al., 2003). In this specific case, the study was carried out in a complex ecosystem, namely the mangrove area, where many factors involved could, in one way or another, modify this evidence. However, an in-depth study on priming in mangroves could well shed light on this established scientific reality.

The significant variation in carbon content in the rhizosphere would be linked to the products of the photosynthesis process released by plants. Indeed, it is known that plants, through the phenomenon of photosynthesis, fix atmospheric CO_2_, using it and producing organic matter (Zhi et al., 2024). The products of photosynthesis are released by root exudation and are discharged underground (Yang et al., 2015). This is used as a key mechanism to reduce CO_2_ in the air and sequester it in the ground. Here, we have been discussing carbon sequestration for years (Shukla et al., 2022).

Phosphorus in its phosphate form is highly insoluble in soils, and it can be bound strongly to oxides of Ca and Fe and to soil organic matter (Chiu et al., 2002). This could explain the reduced concentration of P in non-rhizospheres. However, in rhizospheres, the roots can exude organic acids such as malic and citric acids into the rhizosphere, effectively reducing the pH of the rhizosphere and thus solubilizing the P bound to soil minerals (Sokolova, 2015). Indeed, the presence of calcium, silicon, magnesium, and potassium can improve the mobilization of phosphorus not only by decreasing its sorption capacity but also by promoting the dissolution of phosphate precipitates (Lei et al., 2024). Studies have demonstrated that the joint action of precipitation-dissolution, sorption–desorption, and microbial immobilization-mineralization is generally considered to be the key process that regulates phosphorus mobilization in soil (Cho et al., 2012). Thus, by modifying the soil pH, the phosphorus content and the transformation between the phosphorus bound to different soil elements, such as Fe, Al, and calcium (Ca), can be varied.

Metal speciation in sediment depends on pH and the chemical composition of the plant root exudates (Chiu et al., 2002; Jitendra et al., 2017). Research reported that Mn, Fe oxides, and clays can adsorb cationic heavy metals or bind with them to form co-precipitates. No difference was observed in the concentration of Zn availability in both the rhizosphere and the non-rhizosphere. Additionally, its concentration was extremely low. This can be explained by the fact that this metal can interact easily with other metals such as Fe and Mn. It has been reported that oxides of Fe and Mn can specifically sorb Zn in sediment (Worms et al., 2007). Indeed, the change in redox potential permits the formation of Fe or Mn oxides, which increases the adsorption of Zn. Furthermore, the low concentration of carbon in the non-rhizosphere limited the formation of soluble complexes with Zn. The high availability of K, Mg, and Ca in the mangrove rhizosphere sediment was due to competition for exchange sites. These microelements are known to be highly absorptive and competitive on exchangeable sites (Worms et al., 2007).

### 4.2 Bacterial community abundance and distribution

The main reason for the bacterial variation in different experimental treatments was attributed to the different nutrients available, which in turn are conditioned by the physical and chemical properties of the sediments (Allaouia et al., 2022; Yang et al., 2024). Although it is evident that different soil properties interfere and influence soil microorganisms’ composition and structure, when considering the “depth” factor, the variation is even more considerable between rhizospheres and non-rhizospheres. It was found in this study that in the superficial layers (0–10 cm), the abundance of the bacterial community was much greater. This could be explained by the fact that in the upper layers, the available nutrient concentrations are higher than in the deeper layers. It is obvious that in the rhizosphere vicinity, due to root exudates, nutrients are more abundant (González-López and Ruano-Rosa, 2020; Trevisan et al., 2024), which would result in a considerable abundance of microorganisms in these surroundings. Unlike the rhizospheres, in the non-rhizosphere compartments, and especially in the coastal zone, mineralization, tidal variations, and water runoff upstream of the mangroves would be the origin of the nutrients and their variations (Liu et al., 2020). The unequal distribution of the nutritional resources of bacteria on either side of the rhizosphere and the non-rhizosphere would, in fact, be further evidence supporting a fact already demonstrated, that nutrient abundance is synonymous with microorganism abundance in soil. A positive correlation is therefore established between the various factors and the abundance of bacteria, and this demonstrates that a cause–effect relationship exists between nutrient availability and microorganisms’ biomass.

### 4.3 Changes in the rhizosphere and non-rhizosphere bacterial community and composition

The distribution of soil bacteria supports the reality that microbial distribution in soil depends on where different nutrients are available. Generally, following the notion of the “rhizosphere effect” defined exclusively as the stimulation and creation of favorable conditions for the multiplication and development of soil microorganisms in the vicinity of roots through root exudates, studies tend unanimously to believe that the microbial biomass is more considerable in the rhizospheres than in the bulk sediment (Balyan and Pandey, 2024; Trevisan et al., 2024). It would be reasonable to believe in an abundance of microbes in the rhizosphere rather than on the other side, since it is known that root exudates are an excellent source of nutrients for the development of microbes. However, in this study, although a slight abundance of bacteria was noted in the rhizosphere, there was no significant difference in the abundance of bacteria between the rhizosphere and the non-rhizosphere. This is contradictory to the various scientific reports, which support the fact that the trend is always in favor of the rhizosphere. Nonetheless, three distinct points explain the present observation in order to illuminate our results: (i) selective effects on microorganisms in the rhizosphere zones, (ii) strong competition between rhizosphere bacteria and plants for nutrients, and (iii) competition between bacteria and other microorganisms, particularly fungi present in the rhizosphere area.

Indeed, for the first point, it is important to know that, although root exudates generally increase the biomass of soil microorganisms in the rhizosphere, this may not always be the case under different circumstances. Other scientific research supports a rather flexible opinion that there may be a selective effect on microorganisms in the rhizosphere zones due to various reasons, such as variations in root exudates depending on plant species, soil type, and microbial composition and diversity. For example, Allaouia et al. (2022), DeAngelis et al. (2009), and Mukerji et al. (2006) have reported that the composition of rhizosphere microbes is a function of root quality and composition (DeAngelis et al., 2009; Allaouia et al., 2022). This could therefore lead to a large variation in microbial biomass in the rhizosphere. In this specific case, the study was conducted in mangroves, which are quite special plants, where the excretion of root exudates and the presence of seawater could be significant causes of the variation of nutrients on both sides of the rhizosphere and non-rhizosphere, which would result in variations in microbial abundance between the two experimental groups.

For the second point, it is important to recognize that among the bacteria present in the rhizosphere, there can be a strong competition for nutrients with the plants, which would lead to considerable variation in the bacterial biomass in the rhizosphere (Rengel and Marschner, 2010; Balyan and Pandey, 2024). Studies have shown that in the event of nutrient deficiency, plants adapt in different ways, including morphological modification of the roots, changes in nutrient absorption and transport mechanisms, modification of soil pH, and exudation in general—particularly of organic compounds—to maximize the availability of nutrients (Marschner and Rengel, 2023). These changes in the rhizosphere could therefore affect and cause the bacterial variation observed. Other studies show that most available nutrients are absorbed by the roots in the apical regions, even before the rhizosphere is colonized by microorganisms (Nannipieri et al., 2008). Furthermore, it has also been reported that the mangroves could compete with anammox bacteria for available nitrogen and thus reduce the abundance of anammox bacteria in the deep zone (20–21 cm) (Li et al., 2011). Therefore, the low nutrient concentration in deep areas and the lack of difference between rhizosphere and non-rhizosphere could be attributed to the variation and distribution of root exudates in the mangrove ecosystem.

For the last point, it is clear that another competition between bacteria and other microorganisms, particularly fungi present in the rhizosphere area, could also explain the bacterial variation observed. A study supporting the competition between bacteria and fungi was conducted by Blum et al. (2004). The authors revealed an inverse relationship between the two types of microorganisms and concluded that competition occurs between them.

### 4.4 Ability of specific microbes to tolerate and metabolize pyrene

The concept of evolution and adaptation states that when environmental conditions experience extreme disruptions, it alters the structure of the community of living beings in this ecosystem. However, those with the necessary traits adapt themselves and manage to survive (Rastogi et al., 2012). In this study, pyrene, which is a toxic pollutant, was used as a carbon source for some microorganisms, but at the same time, it can also be toxic for others. Based on the evolutionary theory, only bacteria with the necessary genes conferring tolerance to pyrene stress were present in acclimation samples. This adaptation can be morphological, physiological, and genetic. The appearance of new phylotypes would confirm hypotheses regarding the adaptation response of the bacterial community. The bacterial community in the culture succession was studied using Illumina sequencing of the 16S rRNA gene (Figures 4, 5). We can deduce that the OTUs identified in the majority of the different samples after 30 days of incubation under pyrene stress are functional with respect to pyrene metabolism. This result is similar to those obtained by Wang et al. (2016a) and Hui et al. (2018), showing that changes in the bacterial community in an external stress culture occur over time. The diversity of the bacterial community in the G5 and Gf transfers changed in profile; some taxa that were present in the first generation G1 transfer, such as Thiomonas, Ochrobactrum, Ancylobacter, Defluviimonas, and Hyphomicrobium, progressively disappeared. This can be explained on one hand simply by natural selection, due to the toxicity of pyrene or its metabolites, or on the other hand by the reduction of redundancy during the process of co-acclimation. The isolated OTUs were mainly identified as Burkholderiaceae, Rhodobacteraceae, Bacillaceae, Xanthobacteraceae, Pseudomonadaceae, and Moraxellaceae. These families join the list of families of bacteria involved in, or that may be involved in, the degradation of pyrene. In the final transfer generation (Gf), the results indicated that certain sets of bacteria, “core” OTUs abundant and ubiquitous in the different experimental groups (R, NR, and different depth layers) were identified (Figure 6) and were mainly dominated by the genera *Burkholderia*, *Rhodobacter*, *Bacillus*, and *Pseudomonas*. These bacteria, which were enriched during the acclimation process, are believed to be in one way or another largely responsible for the bacterial responses to the degradation of pyrene. Many of the taxa have been classified as “uncultured”; however, we believe that these bacteria undoubtedly have potential pyrene degradation functions. The fact that they cannot currently be cultivated would simply be evidence that the majority (>99%) of microorganisms in the environment cannot be cultivated in the laboratory.

## 5 Conclusion

This study was carried out with the aim of initially evaluating the diversity and abundance of the bacterial community in mangrove rhizospheres and non-rhizospheres. Secondly, to evaluate the distribution and the influence of nutrients on bacterial diversity and abundance, and finally, to describe the profile of the bacterial community during acclimation under pyrene stress. The results indicate that the unequal relative abundance in both rhizospheres and non-rhizospheres may be the result of the unequal distribution of nutrients on either side, caused by mineralization, immobilization of nutrients in sediments, and especially by the excretion of root exudates. The results of bacterial acclimation under pyrene stress revealed that pyrene had a significant impact on the composition and abundance of the bacterial community. The various phylotypes that appeared under these stressful conditions showed both the stability of the community and the potential for remediation of the polluted environment. Analysis of genetic features within specific acclimatized bacterial species would be one of the future areas of research to illustrate the mechanism of their ability to tolerate or degrade pyrene. Metagenomic, metaproteomic, and metatranscriptomic studies would also reveal co-acclimatization and co-evolution of the bacterial community.

## Data Availability

The data presented in the study are deposited in the NCBI repository, accession number PRJNA827269.
