# Supplementary material for: Bacterial abundance and co-acclimation in mangrove rhizosphere and non-rhizosphere soils under pyrene stress
Source: Front Microbiol. 2026 Feb 6;16:1661315. doi: 10.3389/fmicb.2025.1661315 (PMC12922237; doi:10.3389/fmicb.2025.1661315)

Bacterial Abundance and Co-Acclimation in Mangrove Rhizosphere and Non-Rhizosphere Soils under Pyrene Stress

Han Wang[2†], Ali Mohamed Elyamine[3†], MengTong Liu[2], HongGe Shi[4], Rong Wang[5], Hongzhou Zhang[6], Junfeng Qi[1,2,6*], Wansen Li[1*]

[1] Affiliated Central Hospital of Huanghuai University, Zhumadian, 463000, China

[2] School of Biological and Food Engineering, Huanghuai University, Zhumadian, 463000, China

[3] Department of Life Science, Faculty of Science and Technology, University of Comoros, Moroni 269, Comoros

[4] Zhumadian Academy of Agricultral Sciences, Zhumadian,463000, Henan, China

[5] Zhumadian Tobacco Company of Henan Province, Zhumadian,463000, Henan, China

[6] China Meheco Topfond Pharmaceutical Co., Ltd., Zhumadian, 463000, China.

[†] These authors contributed equally to this work

*** Correspondence:**
20222297@huanghuai.edu.cn (J.F.Q), and zcr3817202@163.com (W.S.L)

Rhizosphere and non-rhizosphere bacterial composition

The single-sample multi-level species composition diagram showed the results of a mangrove rhizosphere/non-rhizosphere single sample, straight from the inside to the outside through multiple concentric circles (Supplementary Fig. 1). From the result of single sample, it can be seen the repartition and the richness in bacteria of mangrove ecosystem. However, naturally, the distribution of bacterial community in rhizosphere and non-rhizosphere seems to be different. This is how the diversity and abundance relative based on phylum and class levels were evaluated and plotted in supplementary Fig. 1 and Fig. 2, respectively.

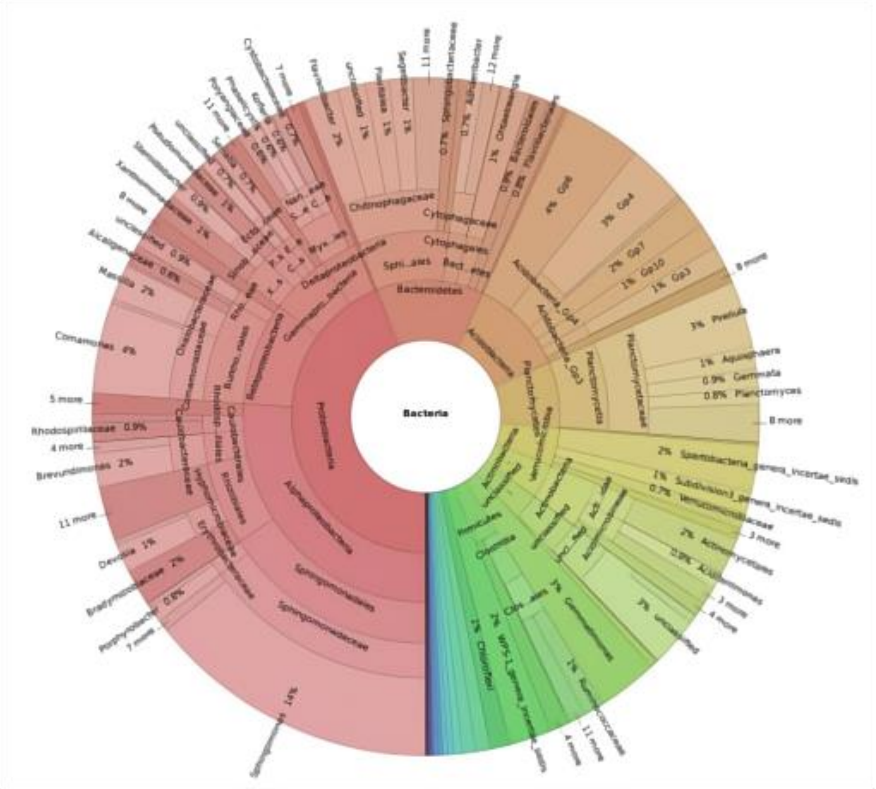

Supplementary Figure 1: The single-sample multi-level species composition diagram shown the results of a mangrove rhizosphere/non-rhizosphere single sample, straight from the inside to the outside through multiple concentric circles.

The relative abundance of bacteria in mangroves rhizosphere and non-rhizosphere was further assessed at the phylum level (Supplementary Fig. 2). The relative abundance of Planctomycetota was significant in non-rhizosphere sample compared to the rhizosphere with respectively, 10.74% and 6.42%. Proteobacteria was the most dominated phylum with 48.84% in rhizosphere against 44.77% in the non-rhizosphere, followed by *Chloroflexi*, *Bacteroidota* and *Desulfobacterota* with respectively 12.67% # 10%, 11.88% # 9.79 and 8% # 5%. By taking into account the depth factor, in the non-rhizosphere, the upper layer (0-5 cm) was more frequented with microbes than the deeper zone (10-20 cm). However, the situation in rhizosphere showed no significant difference.

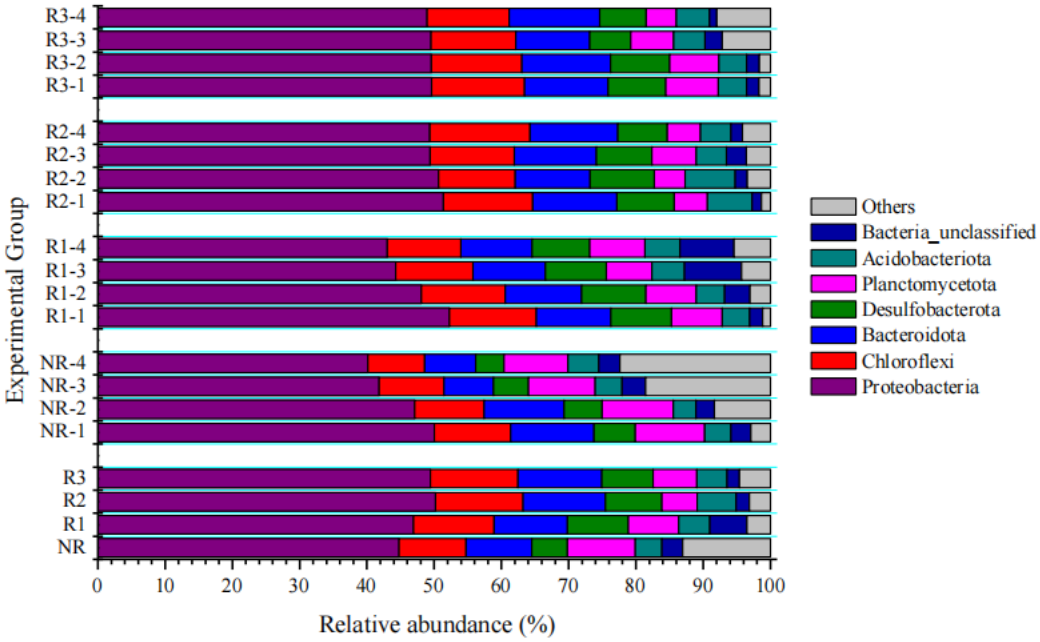

Supplementary Figure 2: Bacterial relative abundance at phylum level. The horizontal and vertical axis respectively represents the name of each sample and the abundance ratio in three replicates. Each color corresponds to phylum name and different color width indicates different abundance. NR = non-rhizosphere, R = rhizosphere

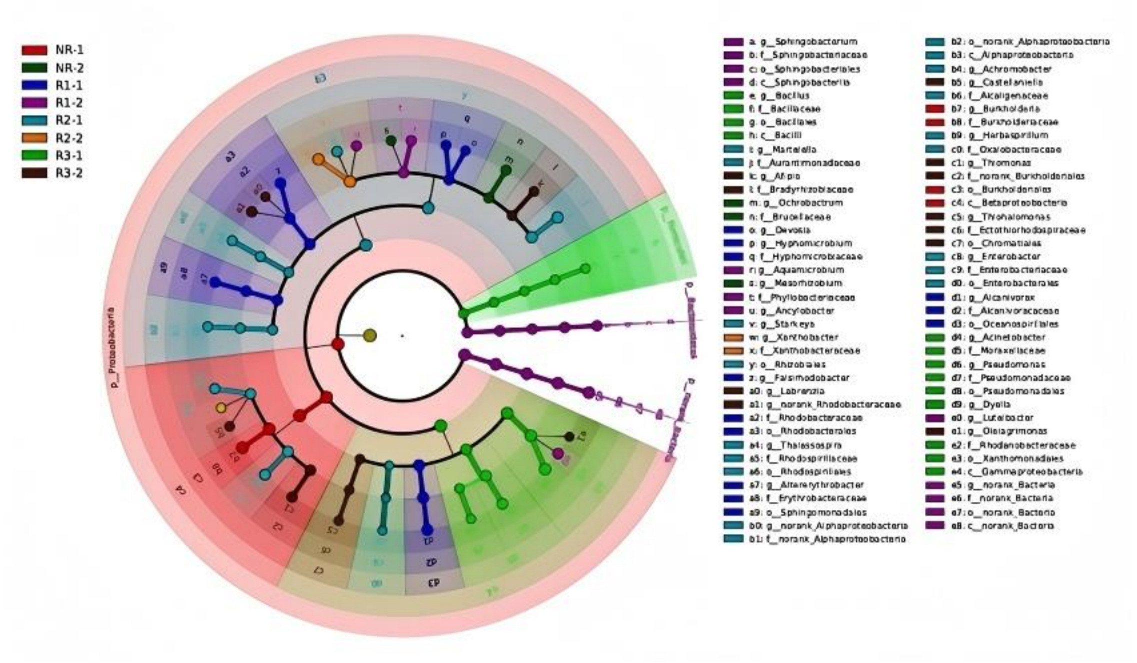

Supplementary Figure 3: Phylogenetic dendrogram of specific bacteria between experimental groups. Different colors represent different groups, and different color nodes in the tree represent the bacterial groups that play an important role in the corresponding color grouping. The yellow nodes represent bacteria that do

not have significant differences from each other. The name represented by the letters of the graph displayed in the legend on the right. Differentiating taxa were detected by the LefSe method analysis ($p < 0.05$). NR = non-rhizosphere, R = rhizosphere

Correlation between bacterial communities in different samples

The graphic in Supplementary Fig. 4 showed the heatmap correlation matrix representing the correlation between different OTUs identified in the different rhizosphere and non-rhizosphere sample. This correlation helps to understand the linear relationship between different identified OTUs in the different experimental group. OTUs identified in rhizosphere sample R2-2 was positively correlated with that of non-rhizosphere sample NR2 (r = 0.93, *p* = 0.007), rhizospheres R3-2 (r = 0.72, *p* = 0.04), R1-1 (r = 0.81, *p* = 0.009), the non-rhizosphere NR1 (r = 0.74, *p* = 0.03), the rhizosphere R1-2 (r = 0.69, *p* = 0.04), and R3-1 (r = 0.62, *p* = 0.04). Interestingly, the identified OTUs in the non- rhizosphere sample NR1 was highly correlated with the non-rhizosphere samples NR2 (r = 0.96, *p* = 0.009).

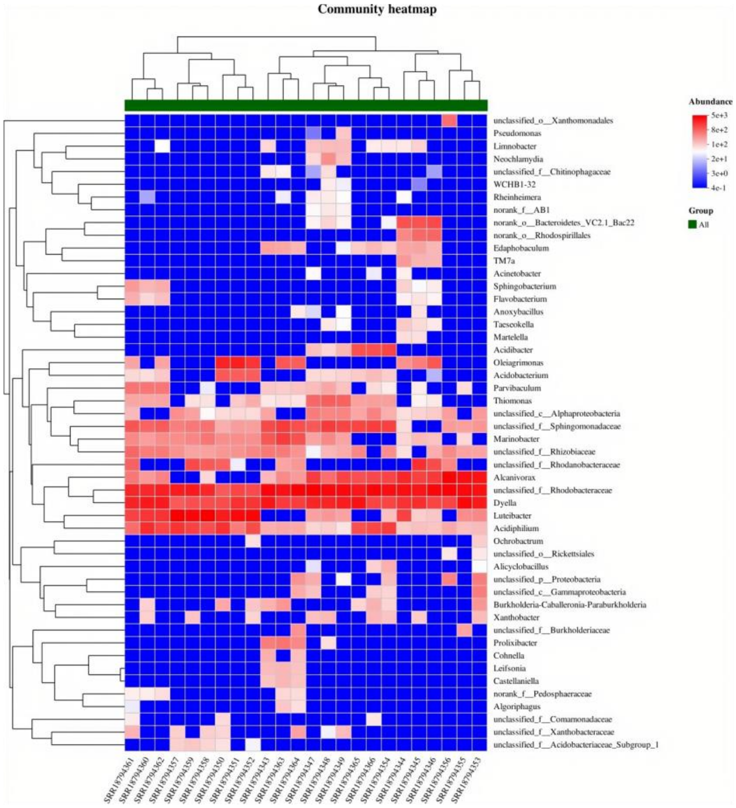

Supplementary Figure 4: the heatmap correlation matrix representing the correlation between different OTUs identified in the different rhizosphere and non-rhizosphere sample. NR = non-rhizosphere, R = rhizosphe

The scatterplot matrix presented in the Supplementary Fig. 5 highlight the correlation between different genera identified in the experimental group during the acclimation process under pyrene stress. The genera *Bacillus* was strongly correlated with, *Pseudomonas* and *Martelella* (r = 0.83, p = 0.03). While *Xanthobacter* was however correlated to *Pseudomonas*, *Martelella*, *Altererythrobacter* and *Sphingobium* (r = 0.86, p = 0.04); the genera *Dylla* was correlated to *Martelella*, *Altererythrobacter* and *Sphingobium* (r = 0.88, p = 0.03). *Altererythrobacter* was positively correlated to *Pseudomonas* and *Sphingobium* (r = 0.81, p = 0.04) and finally, the genera *Sphingobacter* was correlated to *Altererythrobacter* and *Kordiimonas* (r = 0.77, p = 0.04).

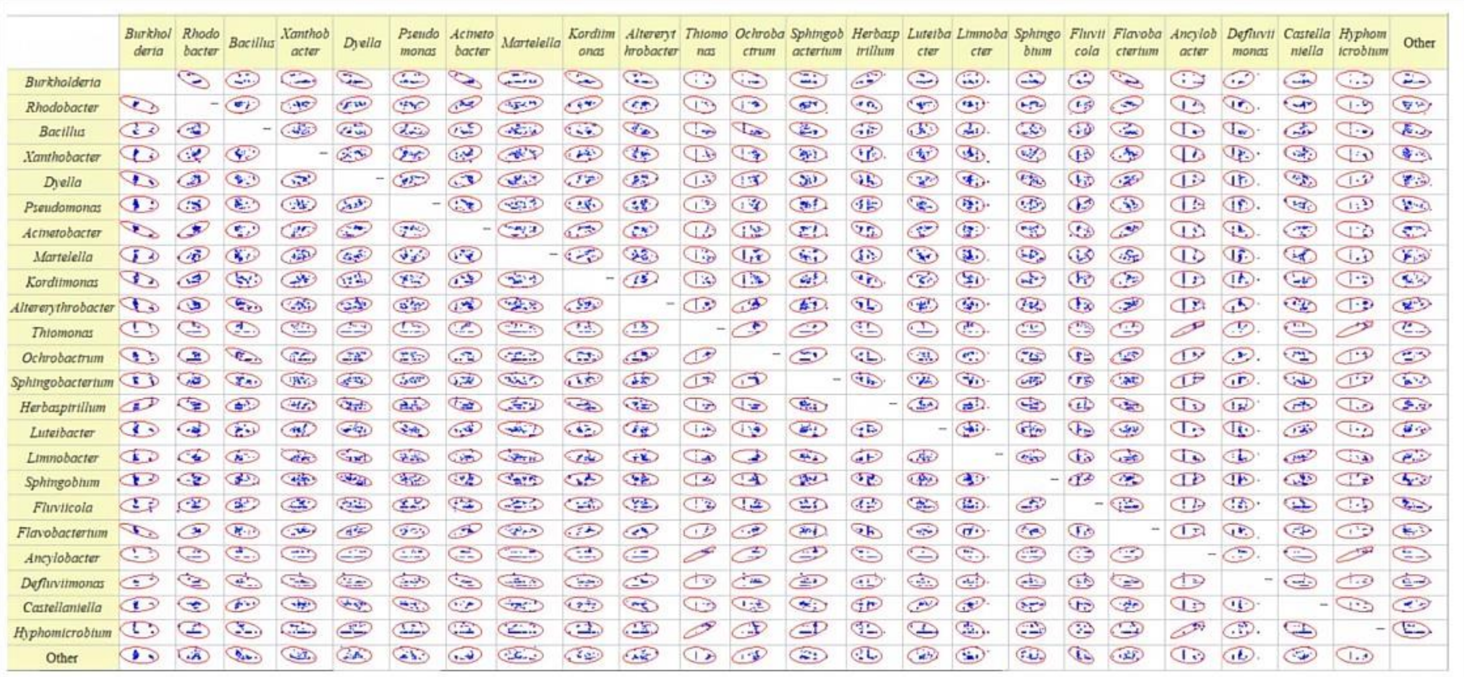

Supplementary Figure 5: The scatterplot matrix presented highlight the correlation between different genera identified in the experimental group during the domestication process under pyrene stress

otu	NR1-1	NR1-2	NR1-3	R1-1	R1-2	R1-3	R2-1	R2-2	R2-3	R3-1	R3-2	R3-3
OTU3	10100	42698	86192	73799	9729	49110	9826	13124	36212	70325	830	2678
OTU22	1859	12	2294	5315	9	12	1768	23	20	12	2294	14977
OTU58	7384	24966	830	34333	939	2365	5249	4068	1352	3962	53391	1191
OTU15	1961	2396	5651	258	162	93	885	437	157	2396	169	258
OTU2	10932	70325	521	46395	23979	29669	4762	3497	2404	42698	521	46395
OTU1	11020	3598	7862	3958	53452	29457	59869	42238	30252	3598	7862	3958
OTU9	42553	324	325	278	166	169	322	219	114	324	325	278
OTU28	819	268	220	325	249	338	276	300	190	268	220	325
OTU49	776	1232	472	104	261	1088	8367	4862	3586	1232	472	104
OTU4	15783	3707	53391	1347	1066	2364	2516	2287	1614	3707	84655	73799
OTU54	35	9	364	1611	13	16	9	8	5	9	364	1224
OTU117	986	492	244	250	102	150	146	181	73	492	244	250
OTU66	2	0	190	0	6120	0	2	1	0	0	190	0
OTU20	252	353	499	53	35	25	4120	149	6347	353	499	53
OTU5	3283	1230	169	714	6768	7643	58040	64771	34250	1230	5651	714
OTU11	8495	2762	101	601	338	507	4567	818	300	2762	101	601
OTU35	770	258	263	400	641	326	196	230	104	258	263	400
OTU19	7567	496	96	343	242	567	647	477	392	496	96	343
OTU27	879	2406	349	1224	987	557	325	323	338	2406	349	1611
OTU7	1189	1751	95	1191	1364	8159	8618	8058	24514	1751	95	34333
OTU41	208	614	88	78	93	145	58	309	46	614	88	78
OTU10	1162	382	119	172	2356	2425	11130	32283	6575	382	119	172
OTU8	7360	254	122	14977	40321	10000	329	819	93	254	122	5315
OTU6	29272	991	101	232	209	375	740	406	275	991	101	232
OTU51	154	47	49	59	46	55	50	52	15	47	49	59
OTU23	225	225	151	19	11	19	22	5402	54	225	151	19
OTU38	138	334	62	128	178	82	43	28	48	334	62	128
OTU87	516	115	26	75	730	10542	162	283	145	115	26	75
OTU45	546	296	157	207	204	177	164	168	432	296	157	207
OTU199	489	412	24	102	74	130	337	79	263	412	24	102
OTU85	983	588	54	409	312	694	632	619	474	588	54	409
OTU65	82	50	10	21	25	48	40	43	20	50	10	21
OTU95	0	0	140	0	0	0	0	0	0	0	140	0
OTU17	1514	232	27	114	626	5445	450	871	1455	232	27	114
OTU12	6441	1004	750	68	1700	2455	2523	633	4951	1004	750	68
OTU16	269	126	20	96	71	149	126	157	98	126	20	96
OTU55	130	131	20	18	35	43	51	38	22	131	20	18
OTU14	221	123	54	71	375	414	43	138	36	123	54	71
OTU46	167	471	45	67	86	62	159	61	27	471	45	67
OTU126	1	5	6	1	2	2	0	1	0	5	6	1
OTU64	404	243	27	180	111	163	150	117	104	243	27	180
OTU84	53	14	21	23	25	18	13	12	9	14	21	23
OTU120	52	22	16	36	39	28	15	21	10	22	16	36
OTU106	102	53	7	38	25	54	49	37	42	53	7	38
OTU111	20	9	8	4	4	7	8	11	1	9	8	4
OTU110	35	13	8	13	9	15	13	5	2	13	8	13
OTU257	123	132	13	86	38	86	88	78	53	132	13	86

OTU43	44	59	82	58	146	22	79	44	42	59	82	58
OTU21	9256	3962	102	935	363	770	2584	7550	834	24966	102	935
OTU70	21	47	14	18	46	8	8	9	12	47	14	18
OTU13	1880	295	27	130	86	173	1896	140	4850	295	27	130
OTU73	167	23	14	22	13	30	33	23	14	23	14	22
OTU40	1342	17	7	7	10	11	16	7	539	17	7	7
OTU29	195	247	6	22	15	30	31	32	2728	247	6	22
OTU160	197	23	29	17	76	124	194	1034	35	23	29	17
OTU247	4	1	1	2	2	2	0	3	0	1	1	2
OTU59	3	61	1	3	12	2	66	1	0	61	1	3
OTU32	450	114	23	71	45	133	1293	1335	132	114	23	71
OTU56	225	242	14	61	41	72	58	43	148	242	14	61
OTU208	49	13	11	15	18	22	16	21	15	13	11	15
OTU34	1423	137	1	3	46	27	3	5	134	137	1	3
OTU52	8	493	1	4	13	30	28	5	69	493	1	4
OTU98	13	68	4	20	10	7	3	3	6	68	4	20
OTU129	20	7	6	12	2	6	2	7	4	7	6	12
OTU26	205	63	12	55	56	461	508	235	2666	63	12	55
OTU122	31	13	2	10	16	27	25	20	13	13	2	10
OTU248	72	27	12	20	19	100	462	299	168	27	12	20
OTU79	44	14	4	12	11	18	25	14	11	14	4	12
OTU62	108	20	20	17	56	45	13	13	13	20	20	17
OTU108	192	114	22	93	120	2573	95	131	58	114	22	93
OTU24	48	21	9	12	36	320	21	20	54	21	9	12
OTU33	1971	238	7	30	34	66	653	54	184	238	7	30
OTU39	50	56	4	12	14	1331	16	7	14	56	4	12
OTU130	5	1	3	2	6	4	0	1	2	1	3	2
OTU18	6180	36	21	73	7812	6492	46	124	219	36	21	73
OTU135	17	11	5	9	2	8	13	6	1	11	5	9
OTU88	222	107	17	85	61	100	93	75	63	107	17	85
OTU74	39	153	15	63	28	31	6	27	18	2	15	63
OTU113	25	8	10	5	6	9	11	12	8	8	10	5
OTU100	7	9	2	2	2	0	3	0	54	9	2	2
OTU203	38	5	2	5	0	4	11	8	5	5	2	5
OTU232	8	3	1	5	4	5	4	9	0	3	1	5
OTU124	17	4	4	8	10	5	3	2	3	4	4	8
OTU175	9	3	2	2	1	2	1	2	1	3	2	2
OTU233	1	1	1	0	1	0	0	0	1	1	1	0
OTU80	12	53	8	25	15	10	6	1	5	53	8	25
OTU176	9	2	3	3	4	4	3	3	0	2	3	3
OTU76	23	46	9	22	16	14	12	16	13	46	9	22
OTU25	149	55	4	28	35	55	5780	85	613	55	4	28
OTU53	51	129	19	58	37	37	19	22	21	129	19	58
OTU127	2	13	1	1	1	1	0	1	0	13	1	1
OTU165	21	4	6	3	6	6	1	10	1	4	6	3
OTU155	11	4	3	5	3	4	6	1	4	4	3	5
OTU194	17	10	8	6	8	8	4	6	8	10	8	6
OTU67	29	37	4	18	28	27	16	9	21	37	4	18

OTU167	14	3	3	3	3	8	2	2	3	3	3	3
OTU114	129	89	6	62	36	91	72	84	43	89	6	62
OTU240	1	0	1	1	5	0	0	0	0	0	1	1
OTU83	77	20	15	37	42	37	16	25	7	20	15	37
OTU104	0	14	13	0	0	2	11	13	9	14	13	0
OTU71	46	51	7	14	12	4	4	13	8	51	7	14
OTU44	84	52	7	31	58	296	67	41	832	52	7	31
OTU188	96	21	1	18	13	13	21	29	13	21	1	18
OTU178	11	1	3	4	3	5	7	4	0	1	3	4
OTU144	2	0	1	1	1	3	0	3	2	0	1	1
OTU69	18	16	19	12	88	17	9	5	17	16	19	12
OTU82	13	54	16	25	48	15	7	9	5	54	16	25
OTU99	333	52	4	24	23	47	55	50	37	52	4	24
OTU68	118	41	4	21	19	24	55	27	16	41	4	21
OTU97	2	24	3	3	5	5	2	0	3	24	3	3
OTU102	1428	21	2	2	3	12	7	16	11	21	2	2
OTU119	22	4	3	4	8	2	11	1	3	4	3	4
OTU156	25	10	2	0	8	6	4	6	2	10	2	0
OTU227	3	2	1	4	1	0	1	2	1	2	1	4
OTU158	22	3	2	0	2	6	0	42	17	3	2	0
OTU132	36	12	6	14	21	13	5	11	6	12	6	14
OTU230	0	1	2	0	0	1	1	2	0	1	2	0
OTU148	95	19	8	118	3	6	15	6	1	19	8	118
OTU146	3	0	2	0	1	3	0	2	1	0	2	0
OTU196	3	2	4	10	0	1	0	2	0	2	4	10
OTU201	3	3	1	3	0	0	1	1	1	3	1	3
OTU121	13	8	4	5	3	9	5	7	4	8	4	5
OTU228	0	1	2	0	0	0	0	0	0	1	2	0
OTU214	11	6	2	5	1	3	1	6	1	6	2	5
OTU209	12	2	2	2	0	3	0	6	3	2	2	2
OTU116	34	8	4	13	9	10	10	13	5	8	4	13
OTU118	1	0	2	3	5	4	0	0	1	0	2	3
OTU141	5	10	7	8	7	5	13	4	11	10	7	8
OTU229	0	1	1	1	0	0	0	1	0	1	1	1
OTU224	5	1	1	0	1	3	0	2	1	1	1	0
OTU173	13	5	3	5	3	4	6	5	6	5	3	5
OTU47	33	229	5	16	173	52	79	19	34	229	5	16
OTU186	10	0	3	0	1	0	0	0	0	0	3	0
OTU103	35	6	4	30	8	11	3	2	6	6	4	30
OTU180	4	3	4	1	0	5	5	1	2	3	4	1
OTU225	6	2	1	1	4	3	3	3	0	2	1	1
OTU217	4	11	1	0	4	1	3	2	0	11	1	0
OTU77	37	15	6	14	6	19	15	21	5	15	6	14
OTU91	64	4	9	3	4	0	2	4	0	4	9	3
OTU187	12	3	7	6	6	0	6	5	1	3	7	6
OTU145	1	2	4	2	3	1	1	1	1	2	4	2
OTU31	42	22	35237	7	3900	499	9	6	1607	22	35237	7
OTU101	3829	19	9	12	8	36	24	23	12	19	9	12

OTU30	20	9	5	10	9	5	5	4	6	9	5	10
OTU136	24	5	2	6	15	10	9	10	2	5	2	6
OTU133	18	8	2	6	12	4	5	9	4	8	2	6
OTU96	10	7	3	6	10	5	7	8	8	7	3	6
OTU109	2	15	4	4	13	11	2	4	6	15	4	4
OTU50	474	12	1	6	6	79	7	7	6	12	1	6
OTU182	12	3	1	5	2	6	6	4	1	3	1	5
OTU142	27	6	4	9	8	9	6	7	4	6	4	9
OTU249	8	3	1	2	1	3	5	5	6	3	1	2
OTU171	0	0	2	1	0	0	0	0	2	0	2	1
OTU36	19	7	3	1220	21	27	7	5	5	7	3	1220
OTU150	0	0	3	2	12	0	0	0	0	0	3	2
OTU202	1	2	1	0	2	1	0	2	0	2	1	0
OTU218	5	1	2	0	1	2	1	1	1	1	2	0
OTU198	4	1	1	0	4	0	1	2	0	1	1	0
OTU72	11	38	6	18	11	5	4	1	3	38	6	18
OTU105	11	5	3	8	27	4	5	4	5	5	3	8
OTU256	0	1	1	2	3	1	2	0	8	1	1	2
OTU123	7	4	1	2	5	6	0	1	3	4	1	2
OTU184	2	0	1	5	0	0	1	0	2	0	1	5
OTU234	19	8	2	82	5	10	3	4	4	8	2	82
OTU163	5	8	1	3	8	3	4	1	5	8	1	3
OTU63	19	12	1	3	37	10	8	10	21	12	1	3
OTU60	21	31	2	4	2	4	5	8	186	31	2	4
OTU251	8	1	1	2	3	2	4	2	2	1	1	2
OTU42	19	6	1	10	679	277	6	3	3	6	1	10
OTU134	9	1	1	0	9	0	3	1	0	1	1	0
OTU210	3	3	1	3	2	6	22	1	6	3	1	3
OTU179	14	11	1	6	1	4	6	5	7	11	1	6
OTU37	12	83	2	1	51	11	6	729	509	83	2	1
OTU92	12	5	1	1	0	4	3	4	65	5	1	1
OTU169	16	3	1	3	5	34	39	43	38	3	1	3
OTU193	3	2	2	1	2	1	1	0	1	2	2	1
OTU195	7	1	1	1	0	0	0	0	0	1	1	1
OTU255	13	1	3	4	1	13	5	6	7	1	3	4
OTU243	2	0	1	2	1	1	1	0	0	0	1	2
OTU149	12	1	1	5	1	5	1	5	2	1	1	5
OTU75	4	4	1	0	2	1	2	0	0	4	1	0
OTU139	12	3	2	1	2	11	3	3	1	3	2	1
OTU78	9	2	1	7	132	37	4	3	1	2	1	7
OTU174	12	4	1	3	4	5	3	4	0	4	1	3
OTU151	17	6	4	3	7	11	7	3	4	6	4	3
OTU128	25	1	1	0	2	3	2	1	1	1	1	0
OTU147	46	29	1	14	11	26	17	20	13	29	1	14
OTU204	4	1	1	1	0	1	0	1	0	1	1	1
OTU192	161	1	1	5	2	2	1	3	0	1	1	5
OTU48	1	0	0	0	1	0	0	1	0	0	0	0
OTU61	10	6	0	2	1	6	5	1	5	6	0	2

OTU81	2	0	0	0	6	11	1	7	0	0	0	0
OTU89	0	3	0	1	4	0	11	1	0	3	0	1
OTU220	3	2	0	1	2	2	0	2	1	2	0	1
OTU157	0	0	0	0	0	0	0	0	0	0	0	0
OTU236	86	2	0	261	586	159	5	9	0	2	0	261
OTU223	8	13	0	8	8	12	10	8	5	13	0	8
OTU153	21	5	0	5	8	9	2	10	4	5	0	5
OTU143	0	3	0	0	2	0	0	0	1	3	0	0
OTU216	6	9	0	4	4	8	6	5	3	9	0	4
OTU239	4	7	0	9	3	4	4	3	3	7	0	9
OTU219	21	7	0	8	4	11	5	8	10	7	0	8
OTU222	11	22	0	4	2	5	3	3	8	22	0	4
OTU112	42	15	0	8	3	11	5	13	9	15	0	8
OTU164	0	2	0	1	0	0	0	0	0	2	0	1
OTU185	14	5	0	5	5	1	6	5	3	5	0	5
OTU260	10	2	0	5	1	3	2	4	2	2	0	5
OTU166	0	1	0	0	0	1	1	1	0	1	0	0
OTU253	3	2	0	3	2	1	4	1	0	2	0	3
OTU183	2	1	0	1	1	1	1	1	0	1	0	1
OTU162	0	0	0	0	1	0	1	1	0	0	0	0
OTU252	3	2	0	2	5	1	2	0	0	2	0	2
OTU177	2	1	0	2	0	2	0	1	0	1	0	2
OTU159	6	0	0	0	0	1	0	0	2	0	0	0
OTU246	4	0	0	3	28	3	0	0	0	0	0	3
OTU250	4	0	0	3	3	4	2	1	3	0	0	3
OTU161	1	0	0	1	0	0	2	0	0	0	0	1
OTU125	3	4	0	3	2	1	2	4	2	4	0	3
OTU170	0	0	0	0	0	0	0	0	0	0	0	0
OTU168	1	1	0	1	0	0	0	1	0	1	0	1
OTU221	3	7	0	5	0	3	0	2	0	7	0	5
OTU191	3	0	0	0	0	0	0	0	0	0	0	0
OTU131	5	3	0	2	1	1	0	1	0	3	0	2
OTU235	0	0	0	34	1	0	1	0	15	0	0	34
OTU57	327	1	0	0	0	0	0	0	1	1	0	0
OTU207	6	1	0	0	0	1	0	0	0	1	0	0
OTU90	33	1	0	1	1	74	17	9	0	1	0	1
OTU172	2	0	0	0	0	0	0	0	0	0	0	0
OTU115	4	0	0	9	25	10	1	1	0	0	0	9
OTU140	9	3	0	4	1	4	2	1	3	3	0	4
OTU241	1	0	0	2	0	1	0	1	0	0	0	2
OTU107	6	1	0	0	0	25	2	64	6	1	0	0
OTU181	3	4	0	0	0	0	0	0	0	4	0	0
OTU190	3	0	0	0	0	1	0	0	0	0	0	0
OTU211	7	1	0	2	1	2	2	3	0	1	0	2
OTU206	11	0	0	0	0	0	0	0	0	0	0	0
OTU189	6	0	0	0	0	0	0	0	0	0	0	0
OTU93	5	77	0	6	13	3	1	0	0	77	0	6
OTU226	8	1	0	2	0	4	1	1	1	1	0	2

OTU137	7	0	0	0	0	0	0	0	0	0	0	0
OTU138	4	2	0	2	1	3	3	1	3	2	0	2
OTU213	3	1	0	0	0	0	1	0	0	1	0	0
OTU197	2	0	0	0	0	0	0	0	0	0	0	0
OTU261	4	2	0	0	1	0	1	0	2	2	0	0
OTU242	3	1	0	3	1	0	0	1	1	1	0	3
OTU212	3	1	0	2	0	0	0	1	1	1	0	2
OTU258	4	2	0	1	1	1	4	1	2	2	0	1
OTU200	8	2	0	2	0	2	2	0	0	2	0	2
OTU237	1	0	0	3	0	0	0	0	0	0	0	3
OTU262	3	2	0	0	0	1	1	2	5	2	0	0
OTU259	2	4	0	1	1	0	4	3	1	4	0	1
OTU238	2	0	0	1	8	1	0	1	0	0	0	1
OTU152	1	0	0	1	16	2	0	0	2	0	0	1
OTU154	0	1	0	0	0	5	0	0	3	1	0	0
OTU86	0	3	0	0	1	0	0	0	66	3	0	0
OTU215	0	1	0	1	0	0	0	1	0	1	0	1
OTU245	0	0	0	1	1	0	1	0	0	0	0	1
OTU244	0	0	0	0	5	0	0	0	0	0	0	0

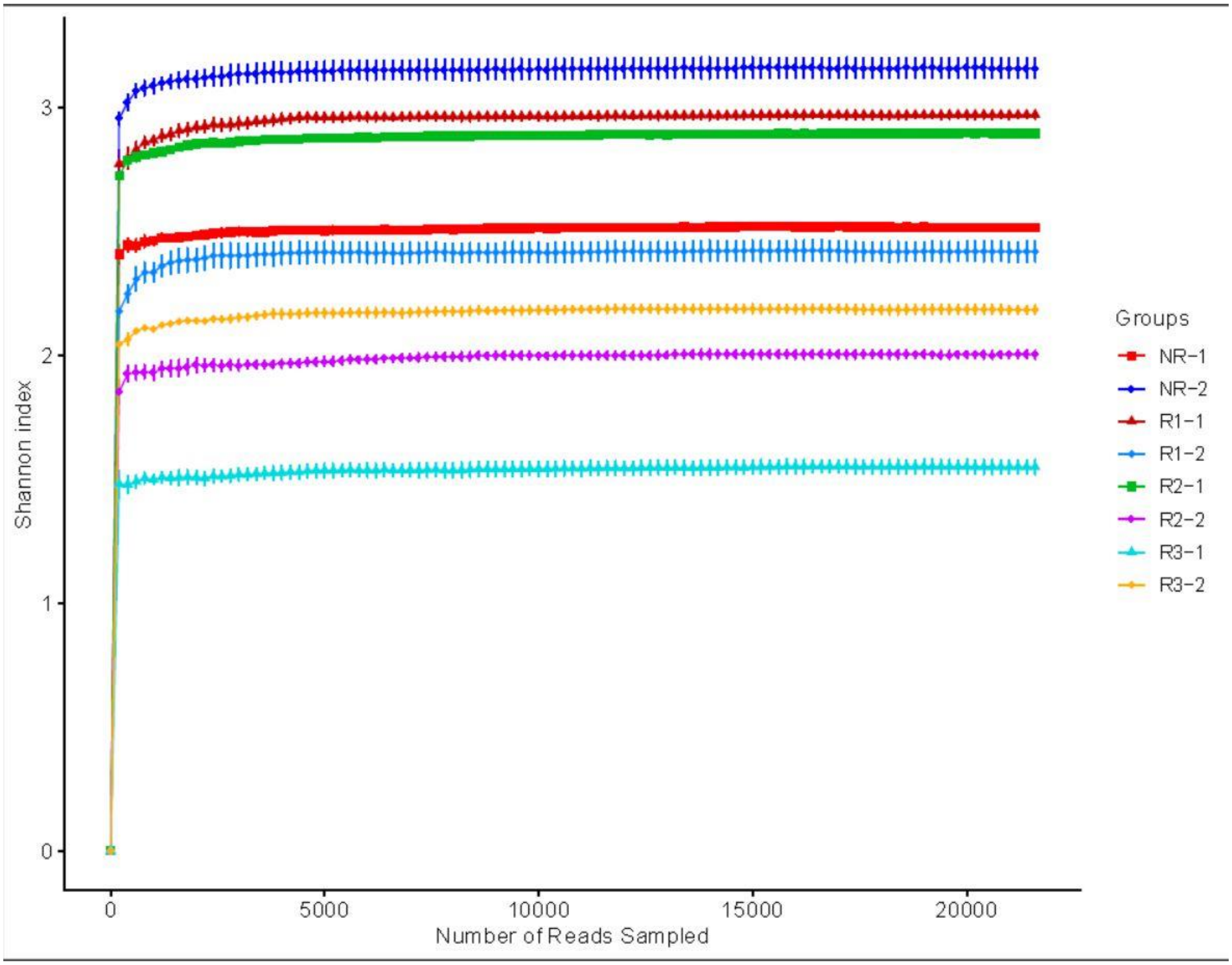

Supplement: Supplementary file 1 [file Data_Sheet_1.pdf]
